# Autism Spectrum Disorders and Purinergic Signaling: A Systematic Review of Emerging Insights from Preclinical Studies

**DOI:** 10.1007/s12031-026-02479-z

**Published:** 2026-02-16

**Authors:** Sonia Guha, David H. Elisha, Rebecca S. Eshraghi, Rahul Mittal, Richard C. Deth, Adrien A. Eshraghi

**Affiliations:** 1 Hearing Research and Communication Disorders Laboratory, Department of Otolaryngology, Miller School of Medicine, University of Miami, Miami, FL USA; 2 College of Pharmacy, Department of Pharmaceutical Sciences, Nova Southeastern University, Fort Lauderdale, FL USA; 3 Diabetes Research Institute, University of Miami, Miller School of Medicine, Miami, FL USA; 4 Department of Neurological Surgery, Miller School of Medicine, University of Miami, Miami, FL USA; 5 Department of Pediatrics, Miller School of Medicine, University of Miami, Miami, FL USA

**Keywords:** Autism spectrum disorder, Purinergic signaling, Neurodevelopmental, Neuroinflammation, Synaptic abnormalities, Behavioral alterations

## Abstract

Autism Spectrum Disorders (ASD), are a group of complex neurodevelopmental conditions characterized by deficits in social communication and the presence of restricted, repetitive behaviors. ASD rates are rising alarmingly in the United States and the reason behind this is obscure. Increasing evidence suggests that purinergic signaling, a form of extracellular signaling mediated by purine nucleosides and nucleotides such as adenosine and adenosine triphosphate (ATP), plays a critical role in neurodevelopment and immune function. This systematic review summarizes preclinical studies focusing on the relationship between purinergic signaling pathways and ASD, focusing on molecular, cellular, and behavioral studies. A comprehensive literature search through 2024 was carried out in PubMed, Scopus, and Web of Science databases following the Preferred Reporting Items for Systematic Reviews and Meta-Analyses (PRISMA) guidelines. A total of 23 preclinical studies met our inclusion criteria and were included in the final review. The findings suggest that aberrant purinergic receptor expression, dysregulated ATP/adenosine status and ectonucleotidase level largely contribute to behavioral and synaptic abnormalities, dysregulation in neurotransmission, neuroinflammation and perturbed glial communication in ASD animal models. These insights support the hypothesis that purinergic signaling dysfunction contributes to the etiology and pathophysiology of ASD and represents a promising therapeutic target.

## Introduction

**Fig. 1:**
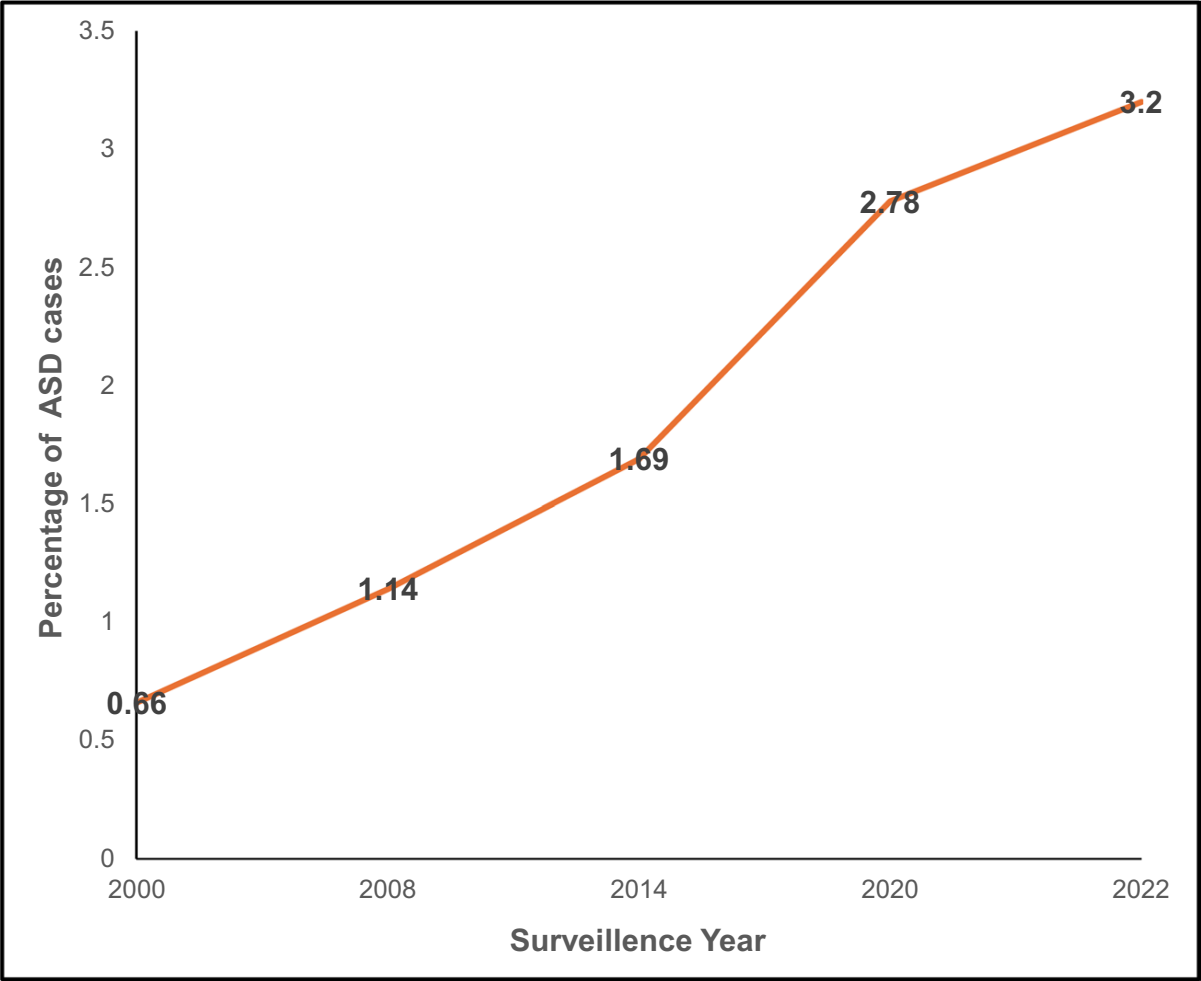
Rising prevalence of ASD in the United States from 2000 to 2020 based on CDC surveillance data. The percentage of ASD cases increased steadily across surveillance years, from 0.66% in 2000 to 3.2% in 2020, highlighting a growing trend in ASD diagnoses over the past two decades

Autism Spectrum Disorder (ASD) is a group of complex neurodevelopmental conditions characterized by deficits in social communication and interaction, behavioral inflexibility, presence of restricted, and repetitive behavioral pattens and atypical response to sensory stimuli (Lord et al. 2020; Zhuang et al. 2024). About 1 in every 31 children were diagnosed with autism in the USA by age 8 in 2022 (3.2%), up from 1 in 150 (0.66%) in 2002 (Fig. 1) (Shaw et al. 2025). This alarming 4.84-fold increase in ASD rates in the last twenty years underscores the urgent need to elucidate its underlying mechanisms and to develop effective therapy for autism. Various genetic, immunological, epigenetic and environmental factors are involved in the etiology of ASD (Zhuang et al. 2024). Over 1450 affected genes have been implicated (https://www.sfari.org/). However, only a small percentage of the disorder is linked with these known mutations, leaving many of the cases to be termed as idiopathic and thereby emphasizing the need to investigate the causes of this disease. Current treatment strategies primarily focus on managing the behavioral symptoms of ASD, highlighting a critical gap in therapies that target the core neurobiological deficits (Kaye et al. 2024).

**Fig. 2:**
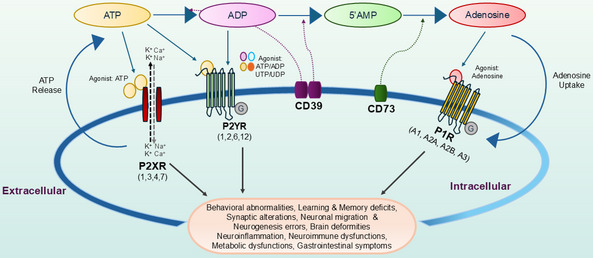
Overview of Purinergic Signaling cascades in Autism. The main component of this system comprises of signaling molecules like purines and pyrimidines, their receptors, and ectonucleotidases. These molecules act on two main types of receptors: (i) P1 receptors (Rs) activated by adenosine. (ii) P2Rs are activated by nucleotides. P2Rs are further subdivided into ionotropic P2X and metabotropic P2Y subtypes. P2XRs are ligand-gated ion channels and respond to ATP while metabotropic P2YRs are G-protein-coupled-receptors (GPCRs) and are activated by ATP, ADP, UTP, and UDP. The ectonucleotidases are ectoenzymes that hydrolyze extracellular nucleotides to the respective nucleosides. The purinergic system has recently emerged as a central player in several pathophysiological conditions like synaptic, neuroinflammatory, behavioral alterations and others in autism spectrum disorders

Neuroinflammation and behavioral abnormalities, the core pathologies of ASD, are regulated by purinergic signaling, comprising signaling molecules like purines and pyrimidines, their receptors, and ectonucleotidases (Fig. 2). Adenosine triphosphate (ATP), a purine, primarily recognized as an energy source, is a key signaling molecule affecting neurobiological functions, along with its metabolites adenosine diphosphate (ADP), adenosine monophosphate (AMP), adenosine, along with pyrimidine nucleotides uridine triphosphate (UTP) and uridine diphosphate (UDP) (Burnstock 1972).

These molecules act on two main types of receptors: (i) P1 receptors (Rs) which are activated by adenosine, are G protein-coupled receptors (GPCRs) comprising four subtypes: A1, A2A, A2B, and A3 (Fredholm et al. 2001). These receptors play diverse neuromodulatory roles including presynaptic inhibition, regulation of the sleep–wake cycle, neuroprotection and neuromodulation of inflammatory responses (Burnstock 2007, 2018; Burnstock et al. 2011) (ii) P2Rs, which are activated by nucleotides and are further subdivided into ionotropic P2X and metabotropic P2Y subtypes (Burnstock and Kennedy 1985). Ionotropic P2XRs are ligand-gated ion channels, with seven subtypes (P2X1-7) (Burnstock 2007, 2018). ATP activates these receptors to induce fast excitatory postsynaptic responses triggered by the influx of cations such as Na^+^ and Ca^2+^, as well as K^+^ efflux. P2XRs are widely distributed in neural and glial cells throughout the central and peripheral nervous systems and are involved in processes such as synaptic transmission and plasticity, inflammation, pain threshold, and communications in the neurons and glial cells (Burnstock 2007, 2018).

Metabotropic P2YRs are also GPCRs, with eight subtypes (P2Y1, 2, 4, 6, 11, 12, 13, 14). These receptors are activated by ATP, ADP, UTP, and UDP, and are involved in a wide array of functions, such as neuroinflammation, presynaptic modulation of neurotransmitter release, neurogenesis, cell proliferation, differentiation, cell death, and neuron-glia interactions and communication (Burnstock 2007, 2018). The delicate balance in signaling mechanisms among the different purines and pyrimidines is tightly controlled by a family of ectonucleotidase enzymes. There are two major types of ectonucleotidases: nucleoside triphosphate diphosphohydrolase-1(CD39) and ecto-5'-nucleotidase (CD73), which play a critical role in regulating purinergic signaling by hydrolyzing ATP and ADP to adenosine. CD39 converts ATP and ADP to AMP, while CD73 further hydrolyzes AMP to adenosine. These enzymes therefore maintain the appropriate balance between ATP and adenosine in the extracellular space and modulate activity of their corresponding receptors (Antonioli et al. 2013).

Given the wide distribution and functional roles of the purinergic signaling system in basic neurodevelopmental processes, it is plausible that its dysregulation has broad and significant effects on brain development and is associated with multiple ASD-related abnormalities.

## Methods

### Search Strategy and Protocol Registration

This systematic review was conducted following the guidelines outlined in the Preferred Reporting Items for Systematic Reviews and Meta-Analyses (PRISMA) statement. The review protocol was developed a priori and registered in the International Platform of Registered Systematic Review and Meta-Analysis Protocols (INPLASY) [Registration number: INPLASY202550091].

A comprehensive literature search was conducted to identify studies evaluating the role of purinergic signaling in ASD. The search was carried out in four major electronic databases: PubMed, Scopus, Embase, and Web of Science. The search covered studies published through 2024 and utilized a combination of Boolean operators, Medical Subject Headings (MeSH), and database-specific keywords to ensure a comprehensive and focused retrieval of relevant literature.

The search strategy used was as follows:

(“Autism Spectrum Disorder”[MeSH] OR “Autism” OR “Autistic Disorder” OR “ASD” OR “Pervasive Developmental Disorder” OR “Neurodevelopmental Disorder”)

and

(“Purinergic Signaling”[MeSH] OR “Purinergic System” OR “ATP Signaling” OR “Adenosine Receptors” OR “Purinergic Receptors” OR “P2X Receptors” OR “P2Y Receptors” OR “P1 Receptors” OR “Extracellular ATP” OR “Purinergic Modulation”)

Equivalent search strategies were adapted for use in Scopus, Embase, and Web of Science.

### Study Selection

Studies were included, if they investigated the role of purinergic signaling in pathophysiology, diagnosis, or potential treatment of ASD. Eligible studies included original peer-reviewed articles involving preclinical models that provided empirical data on purinergic receptors, extracellular ATP, or related signaling mechanisms in the context of ASD or autism-like behavior. Both observational and interventional study designs were eligible.

Exclusion criteria included review articles, editorials, conference abstracts, case reports without mechanistic data, and studies that did not directly assess purinergic signaling in relation to ASD or neurodevelopmental phenotypes.

All records retrieved during the database search were imported into Covidence, a web-based tool used for managing systematic reviews. Duplicate records were removed, and two reviewers independently screened titles, abstracts, and full texts according to the inclusion and exclusion criteria.

### Data Extraction

Data was independently extracted by two reviewers (SG, DE) using a standardized data extraction form. Extracted data included study design, population or model type, experimental approach, purinergic targets investigated (e.g., P2X, P2Y receptors), key findings, and outcome measures relevant to ASD phenotypes (e.g., social behavior, repetitive behaviors, neuroinflammation). In cases of disagreement, a third reviewer was consulted to resolve discrepancies.

### Risk of Bias and Quality Assessment

Risk of Bias (ROB) was assessed using tools appropriate to study design. The Systematic Review Center for Laboratory Animal Experimentation (SYRCLE ROB tool) was applied (Hooijmans et al. 2014). Two reviewers independently assessed each study, and any disagreements were resolved through discussion or adjudication by a third reviewer.

### Data Synthesis

Data included in the studies were reported in both narrative and tabular formats. Studies were grouped by study type (genetic modifications vs environmental exposures) and animal model type (e.g. different knockout animal models, environmental agents). Findings were summarized to highlight the preclinical models of ASD, components of the purinergic signaling system that were dysregulated in the preclinical models, the mechanisms that were affected, therapeutic implications, and limitations of the animal models studied.

## Results and Discussion

### Study Selection

**Fig. 3:**
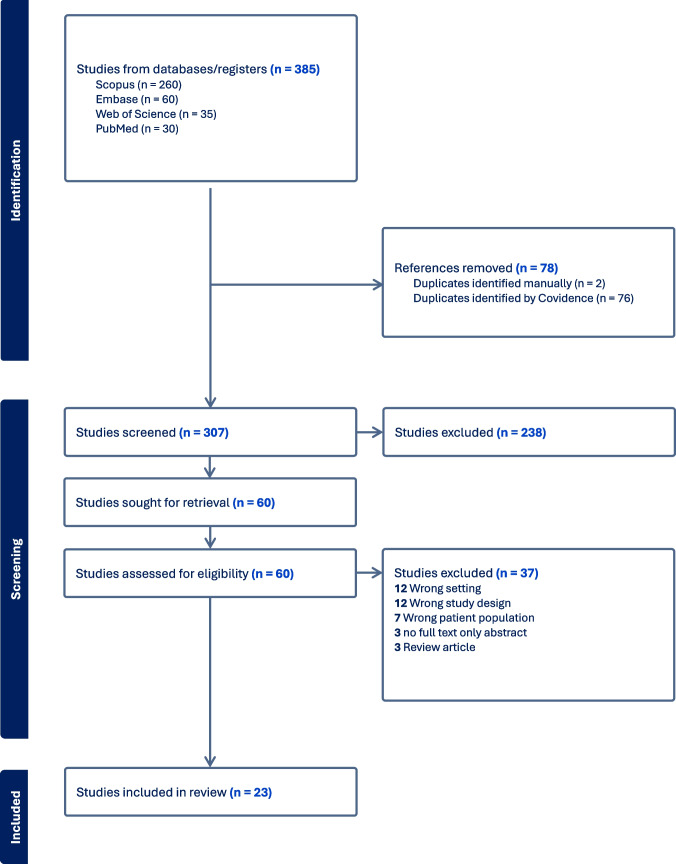
PRISMA flow diagram depicting study. This figure represents a PRISMA (Preferred Reporting Items for Systematic Reviews and Meta-Analyses) flow diagram, showing the process of study selection discussed in this systematic review

A total of 385 articles were initially identified. Of these, 60 studies were selected after excluding 238 duplicates or irrelevant articles. Following the full-text review, a total of 23 articles were selected for this study after excluding 37 studies for reasons outlined in the PRISMA diagram, such as outcomes not relevant to this study or ASD. The PRISMA flow diagram depicting this study is shown in Fig. 3.

### Quality Assessment of Included Studies

**Fig. 4:**
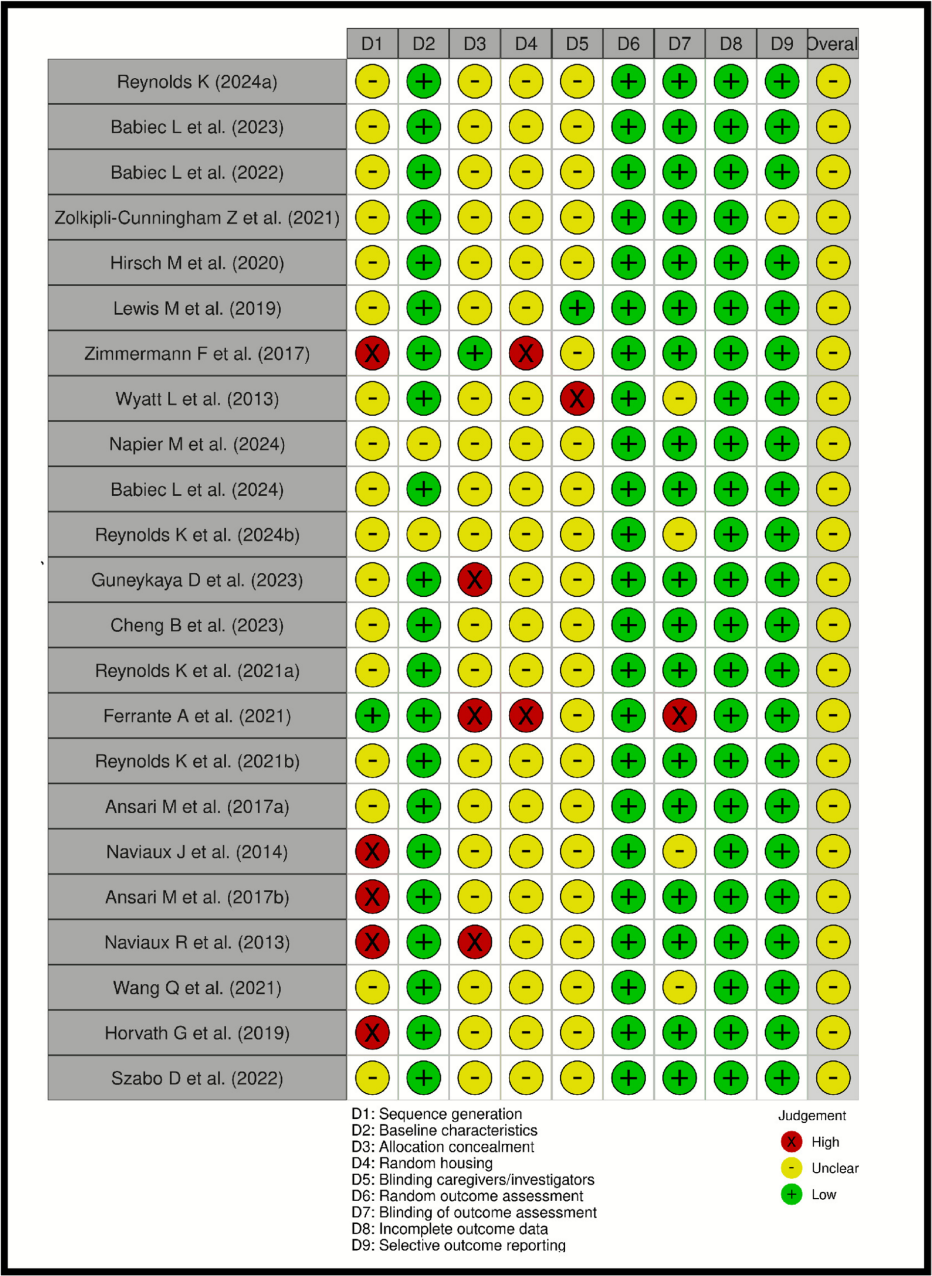
Risk of bias assessment for included preclinical studies evaluating purinergic signaling in autism spectrum disorder. Each study was assessed across nine domains using the SYRCLE risk of bias tool. Green circles indicate low risk of bias, yellow circles indicate unclear risk, and red circles indicate high risk. D1–D9 correspond to sequence generation, baseline characteristics, allocation concealment, random housing, blinding of caregivers/investigators, random outcome assessment, blinding of outcome assessment, incomplete outcome data, and selective outcome reporting

The critical review of the studies included in this systematic review is shown in Fig. 4.

### Data Extraction and Study Outcomes

A summary of the main findings of studies included in this review article is shown in Table 1. The studies included in this review reflect different aspects of autism in rodents and zebrafish and can be broadly categorized into:

1. Genetic modifications: involving mutations in specific genes known to be associated with ASD in humans (12 studies)
2. Environmental exposures:involving prenatal or postnatal exposure to environmental risk factors on neurodevelopment (11 studies)

**Table 1:** Summary of preclinical studies on purinergic signaling in autism spectrum disorders

Studies	Year	Title	Main findings	Model used
Wyatt L et al. (Wyatt et al. 2013)	2013	Sociocommunicative and sensorimotor impairments in male P2X4-deficient mice	P2X4R KO mice showed neurodevelopmental impairments	Mouse model of neurodevelopmental disease—*P2X4-/-* mouse
Naviaux R et al. (Naviaux et al. 2013)	2013	Antipurinergic therapy corrects the autism-like features in the poly(IC) mouse model	Suramin alleviates behavioral symptoms in MIA-ASD mice	Mouse model of Autism—MIA model
Naviaux J et al. (Naviaux et al. 2014)	2014	Reversal of autism-like behaviors and metabolism in adult mice with single-dose antipurinergic therapy	Single-dose antipurinergic therapy reversed ASD-like phenotypes	Mouse model of Autism—MIA model
Zimmermann F et al. (Zimmermann et al. 2017)	2017	Analysis of Extracellular Nucleotide Metabolism in Adult Zebrafish After Embryological Exposure to Valproic Acid	VPA exposure altered biochemical and molecular purinergic parameters	Zebrafish model of Autism—Zebrafish embryos exposed to VPA
Ansari M et al. (Ansari et al. 2017b)	2017	Adenosine A2A receptor modulates neuroimmune function through Th17/retinoid-related orphan receptor gamma t (RORgammat) signaling in a BTBR T(+) Itpr3(tf)/J mouse model of autism	A2AR regulates cytokine production in BTBR mouse	Mouse model of Autism—BTBR mouse
Ansari M et al. (Ansari et al. 2017a)	2017	Activation of adenosine A2A receptor signaling regulates the expression of cytokines associated with immunologic dysfunction in BTBR T(+) Itpr3(tf)/J mice	A2AR modulates cytokine secretion restoring immune balance	Mouse model of Autism—BTBR mouse
Lewis M et al. (Lewis et al. 2019)	2019	Reduction of repetitive behavior by co-administration of adenosine receptor agonists in C58 mice	Adenosine A1 and A2A receptor agonists reduced repetitive behavior	C58 mouse model for repetitive behaviour
Horva´th G et al. (Horvath et al. 2019)	2019	P2X7 Receptors Drive Poly(I:C) Induced Autism-like Behavior in Mice	Activation of the purinergic ion channel P2X7 (P2X7R) is necessary and sufficient to transduce autism-like features in MIA male offsprings	Mouse model of Autism—MIA and *P2X7-/-* mouse
Hirsch M et al. (Hirsch et al. 2020)	2020	Effects of single-dose antipurinergic therapy on behavioral and molecular alterations in the valproic acid-induced animal model of autism	Effects of suramin, a purinergic antagonist, on behavioral and molecular features in VPA model	Autism rat model—Prenatal exposure to VPA
Zolkipli-Cunningham Z et al. (Zolkipli-Cunningham et al. 2021)	2021	Metabolic and behavioral features of acute hyperpurinergia and the maternal immune activation mouse model of autism spectrum disorder	Characterized features in hyperpurinergia and maternal immune activation mice models	Mouse model of Autism—Hyperpurinergia and MIA model
Reynolds K et al. (Reynolds et al. 2021a)	2021	Converging purinergic and immune signaling pathways drive IL-6 secretion by Fragile X cortical astrocytes via STAT3	P2Y signaling drives pro-inflammatory IL-6 in Fmr1 KO astrocytes	Mouse model of Fragile-X Syndrome (FXS)—Fmr1 KO mouse
Ferrante A et al. (Ferrante et al. 2021)	2021	Adenosine A(2A) receptor inhibition reduces synaptic and cognitive hippocampal alterations in Fmr1 KO mice	A2AR antagonism normalizes synaptic and cognitive alterations	Mouse model of FXS—*Fmr1-/-* mouse
Reynolds K et al. (Reynolds et al. 2021b)	2021	Adenosine A(2A) receptor inhibition reduces synaptic and cognitive hippocampal alterations in Fmr1 KO mice	Suramin blocks P2Y2 and P2Y6 upregulation in astrocytes	Mouse model of FXS—*Fmr1-/-* mouse
Wang Q et al. (Wang et al. 2021)	2021	Impaired calcium signaling in astrocytes modulates autism spectrum disorder-like behaviors in mice	ATP-P2X2R signaling drives ASD behavior in IP3R2-deficient mice	Mouse model of Autism—*IP3R2-/-* mouse and astrocyte-specific IP3R2 conditional KO mouse
Babiec L et al. (Babiec et al. 2022)	2022	Prenatal exposure to valproic acid induces alterations in the expression and activity of purinergic receptors in the embryonic rat brain	Changes in expression and activity of purinergic receptors during brain development in VPA model	Rat model of Autism—Exposure to VPA
Szabo´ D et al. (Szabo et al. 2022)	2022	Maternal P2X7 receptor inhibition prevents autism-like phenotype in male mouse offspring through the NLRP3-IL-1β pathway	The P2X7/NLRP3-IL-1β pathway regulates MIA-induced inflammation and autism in male offsprings	Mouse model of Autism—MIA and *P2X7-/-* mouse
Babiec L et al. (Babiec et al. 2023)	2023	Alterations of Purinergic Receptors Levels and Their Involvement in the Glial Cell Morphology in a Pre-Clinical Model of Autism Spectrum Disorders	VPA exposure induces glial cell activation and purinergic signalling imbalance in adolescent rat brains	Rat model of Autism—Exposure to VPA
Guneykaya D et al. (Guneykaya et al. 2023)	2023	Sex-specific microglia state in the Neuroligin-4 knock-out mouse model of autism spectrum disorder	Disrupted P2Y12 signaling in NLGN4-/- male mouse microglia	Mouse model of Autism—*NLGN4-/-* mouse
Cheng B et al. (Cheng et al. 2023)	2023	Vitamin A deficiency from maternal gestation may contribute to autistic-like behaviors and gastrointestinal dysfunction in rats through the disrupted purine and tryptophan metabolism	Vitamin A deficiency causes autistic-like behavior via purine metabolism	Rat model of Autism—Vitamin A deficient rats
Reynolds K et al. (Reynolds et al. 2024a)	2024	Purinergic Signalling Mediates Aberrant Excitability of Developing Neuronal Circuits in the Fmr1 Knockout Mouse Model	Showed that P2Y2 purinergic receptors play a distinct role in pathological Fragile X Syndrome (FXS) neuronal activity	Mouse model of Fragile X Syndrome (FXS)—*Fmr1-/-* mouse
Napier M et al. (Napier et al. 2024)	2024	P2X7 expression patterns in the developing Fmr1-knockout mouse hippocampus	Altered P2X7R expression during development in Fmr1 KO mouse	Mouse model of FXS—*Fmr1-/-* mouse
Babiec L et al. (Babiec et al. 2024)	2024	Correction: Babiec et al. Alterations of Purinergic Receptors Levels and Their Involvement in the Glial Cell Morphology in a Pre-Clinical Model of Autism Spectrum Disorders	P2 receptor antagonists mitigated ASD-like behavioral and synaptic changes	Rat model of Autism—Prenatal exposure to VPA
Reynolds K et al. (Reynolds et al. 2024b)	2024	Dysregulated Purinergic Signalling in Fragile X Syndrome Cortical Astrocytes	Purinergic dysregulation demonstrated in FXS cortical astrocytes	Mouse model of FXS—*Fmr1-/-* mouse

### Studies on Genetic Modifications and Purinergic Signaling Dysregulations

*Fragile X messenger ribonucleoprotein 1 (Fmr1)—*Knockout (KO), *(Fmr1-/-)* mouse model (6 Studies): Mutations in the *FMR1* gene lead to Fragile X syndrome (FXS), a monogenic disease with ASD phenotypes including neurodevelopmental and intellectual disabilities as well as neuro inflammatory symptoms (Richter and Zhao 2021). The *Fmr1-/-* mouse model is used to study FXS and ASD (Bernardet and Crusio 2006). The most important behavioral alterations observed in the *Fmr1*-/- mouse are social deficits, increased repetitive behavior, anxiety and hyperactivity (Bernardet and Crusio 2006).

In 2021 Reynolds et al. sought to understand if astrocyte-mediated purinergic signaling is dysregulated in Fragile X Syndrome (FXS) (Reynolds et al. 2021b). This is an important issue, since purinergic signaling is one of the most ubiquitous signaling systems for glial-neuronal and glial-glial crosstalk. The study showed that in the presence of exogenous ATP and UTP the intracellular calcium responses were elevated in *Fmr1* KO astrocytes compared to wild-type (WT) controls. Furthermore, the expression of purinergic P2Y2 and P2Y6Rs was elevated in *Fmr1-/-* astrocytes, both in vitro and in dissociated cortical tissues. Together these findings point to an upregulation of purinergic signaling pathways in the absence of the *Fmr1* gene. Suramin, a nonspecific P2Y antagonist, normalized this response. The expression and secretion of Thrombospondin-1 (TSP-1) a synaptogenic protein regulated by P2YR activation, also increased in *Fmr1-/-* astrocytes while its levels were transiently elevated in the cortex of the *Fmr1-/-* mouse. In summary, activation of P2Y2/P2Y6R-mediated purinergic signaling in *Fmr1-/-* mouse cortical astrocytes promoted aberrant excitatory synaptic transmission in the cortex. Targeting this cortical purinergic signaling may offer a potential therapeutic approach to restore normal excitability and improve cognitive and behavioral outcomes in affected individuals with FXS and ASD.

In a follow up study in 2021 Reynolds et al. investigated the mechanisms underlying elevated interleukin (IL)−6 secretion in cortical astrocytes from the *Fmr1-/-* mouse (Reynolds et al. 2021a). The group explored how purinergic and immune pathways coordinated to secrete IL-6, a pro-inflammatory cytokine important for neuroinflammation and synaptic alteration. The study demonstrated that activation of glial P2Y purinergic receptors enhanced the secretion of glycoprotein tenascin C (TNC) and promoted phosphorylation of transcription factor, signal transducer and activator of transcription 3 (Stat3). Phosphorylated Stat3 (pStat3) increased gene expression of IL-6 and exacerbated pro-inflammatory responses in the FXS-mouse cortex. Stat3 knockdown and Toll-like receptor 4 (TLR4) antagonism (with TAK242) normalized IL-6 release, suggesting that purinergic and immunological pathways converge to drive IL-6 secretion via Stat3. A relatively recent study by the same group showed that upregulated purinergic signaling in cortical FXS astrocytes increases neural firing rates (Reynolds et al. 2024a). The increased firing was, however, associated with reduced synchrony, suggesting a disruption in coordinated neural activity. These findings underscore the significance of astrocyte-mediated purinergic signaling in the development of neural circuitry and point to its potential role in the pathophysiology of FXS. Given the frequency of synaptic dysfunction observed in ASD, such preclinical studies focusing on the effects of purinergic signaling on synaptic functioning and neurodevelopment are of paramount importance. A selective P2Y2R antagonist, AR–C 118925XX further restored this aberrant neural activity in *Fmr1-/-* neural cultures, highlighting that P2Y2 is a potential therapeutic target for FXS. Targeting P2Y2R-mediated purinergic signaling pathways may, therefore, offer a promising therapeutic strategy for developmental intellectual disabilities associated with ASD.

In a separate study in 2024, Napier et al. investigated the role of purinergic P2X7R’s in hippocampal brain development in the FXS mouse model (Napier et al. 2024). Their research showed that P2X7 expression was decreased in the hippocampus of *Fmr1-/-* mouse at postnatal (P) days 14 and 21, critical for neurite outgrowth and synaptic refinement. This suggests that altered hippocampal P2X7 expressions during postnatal stages may affect brain development in the FXS mouse. Immunohistochemical analysis further revealed increased expression of P2X7Rs by *Fmr1* KO microglia, indicating glial inflammation and a shift in purinergic signaling within the hippocampal microenvironment. This study also found sex-dependent variations in P2X7R expression. Male *Fmr1-/-* mice exhibited reduced colocalization of P2X7R with the neuronal cells at P14 and P21 compared to females. Conversely, only female *Fmr1-/-* mouse showed a reduction in neuronal P2X7R expression in the dorsal hippocampus, highlighting the importance of considering sex as a variable in purinergic signaling and autism research.

In the same year, Reynolds et al. reported dysregulation in the extracellular concentration of adenosine-based purinergic molecules like UDP, ATP, AMP, and intracellular adenosines in *Fmr1-/-* astrocytes (Reynolds et al. 2024b). Conditioned media from KO astrocytes had a higher concentration of secreted adenosine. The *Fmr1-/-* astrocytes also had higher levels of active glycosylated membrane-bound CD39 ectonucleotidases. Alterations in their level, together with a change in the adenosine-based purine metabolites in *Fmr1-/-* astrocytes, points to an impairment in purine metabolism and signaling which could contribute to the synaptic and network dysfunctions characteristic of FXS.

Taken together, the above two studies demonstrate that absence of astrocyte Fragile X messenger ribonucleoprotein (Fmrp) in a preclinical ASD-model (*Fmr1-/-* mouse), leads to widespread purinergic dysregulation affecting not only receptor expression but also the production and availability of several purines which are associated with improper brain development in ASD.

A study published by Ferrante et al. in 2021 described the interaction of adenosine A2A receptors (A2ARs) and Fmrp in the cortex and hippocampus of *Fmr1* WT/KO mice suggesting that Fmrp regulates A2AR expression during early neurodevelopment (Ferrante et al. 2021). Extracellular electrophysiology experiments in brain slices of *Fmr1-/-* mouse showed that A2ARs and metabotropic glutamate 5 receptors (mGlu5Rs) exhibit a synergistic interaction in the hippocampus, which is enhanced in *Fmr1-/-* mice. A2AR agonists and antagonists modulated hippocampal mGlu5R-induced synaptic effects/depression in *Fmr1* WT and KO.

mice and the ability of the A2AR agonist CGS21680 to modulate this interaction was greater in absence of Fmrp, suggesting a greater synergistic coupling between A2AR and mGlu5Rs in the *Fmr1-/-*. Furthermore, the pharmacological blockade of A2ARs by the orally available antagonist, istradefylline, reduced the synaptic alterations and dendritic spine density in *Fmr1-/-* mouse. Learning and memory deficits in *Fmr1-/-* were also corrected by istradefylline, probably through synaptic modifications and reduction in dendritic spine density. This beneficial effect of istradefylline is encouraging, since FXS patients suffer from cognitive disabilities. Apart from these behavioral effects, the study also delineated the downstream mechanistic pathway of the A2ARs. Istradefylline ameliorated mTOR/mGlu5R hyper-phosphorylation and restored levels of the truncated form of tropomyosin receptor kinase B (TrkB). Truncated TrkB are important regulators of impaired BDNF signaling and have been identified as pivotal contributors to the development of several neurological disorders. These receptors lack intrinsic tyrosine kinase activity, resulting in signaling mechanisms that differ from those of their full-length counterparts (Tessarollo and Yanpallewar, 2022). The full-length TrkB protein supports signaling for brain-derived neurotrophic factor (BDNF) and striatal-enriched protein tyrosine phosphatase (STEP) within the hippocampus. Collectively, these findings suggest that istradefylline exerts its effects via mTOR/mGlu5R and TrkB/BDNF pathways. RNA immunoprecipitation studies further showed that A2AR-mRNA is associated with the RNA-binding protein, Fmrp, in a complex in the cortex and hippocampus. This suggests that during early stages of neurodevelopment, Fmrp could regulate translation of A2AR mRNA. Therefore, abnormalities related to A2AR deficiency could start early in development in *Fmr1-/-* mice. Overall, this study demonstrated that blocking the purinergic A2AR, could restore mGlu5R-mediated effects in FXS including synaptic and cognitive alterations associated with this condition.

#### Limitations

The *Fmr1-/-* mouse model typically uses a full genetic knockout while in humans frequently there is a silencing of the gene that leads to a deficiency of the protein, rather than a complete absence (Bakker and Oostra 2003). So, the relevance of findings in this model to human FXS must be interpreted with caution. The reported studies lack vivo/across model validation and focus on one cell type/pathway, with challenges in quantifying ligand levels, metabolites and limited functional, behavioral linkage. They capture a snapshot rather than full developmental dynamics. These constraints limit generalizability to the whole intact brain and full developmental trajectory of purinergic signaling in this model.

*BTBR T + Itpr3tf/J (BTBR) mouse model (2 Studies):* The *BTBR* mouse, originally bred for diabetic research studies, has deletions in the inositol triphosphate receptor 3 gene (Itpr3) which affects its social communication to food-preference. This mouse displays strong and consistent autism-related behaviors broadly classified under (1) Social deficits like impaired social interactions, less social play and poor performance in three-chamber social interaction tests. (2) Communication deficits demonstrated by reduced ultrasonic vocalizations and altered vocal pattens during social interactions (3) Repetitive behaviors like increased self-grooming, repetitive jumping, anxiety and displaying behavioral inflexibility in tasks requiring cognitive flexibility. (4) Neurological and immune features displayed by altered brain morphology, errors in axon guidance and neurogenesis as well as synaptic and neuroimmune dysfunctions (Meyza et al. 2013). Considering its complex genetic, molecular and physiological background, the *BTBR* mouse represents an idiopathic form of autism and supports a model to study multiple aberrations found throughout the ASD population in one animal (Meyza and Blanchard 2017; Meyza et al. 2013).

Ansari et al. used the *BTBR* ASD mouse model to investigate if P1 purinergic A2AR regulation of neuroimmune function through the retinoid-related orphan receptor gamma (RORγt)/Th17 signaling pathway (Ansari et al. 2017b). The A2AR agonist (CGS 21680) and an antagonist (SCH 5826) were administered for 7 days to evaluate behavioral outcomes, such as self-grooming, repetitive behaviors, pain sensitivity, and immune responses. The study revealed that activation of A2ARs with CGS 21680 reduced pro-inflammatory markers IL-17A, RORγt, Stat3, and pStat3 and elevated anti-inflammatory markers [forkhead box P3 and IL-10] in both spleen CD4 + T cells and brain tissues of the BTBR mouse as compared to the C57BL/6 (B6) WT. Conversely, blocking A2ARs with SCH 5826 produced opposite effects. These results suggest that A2AR activation may have therapeutic potential in addressing the neuroimmune dysfunction frequently associated with ASD. This study also illustrates how purinergic dysregulation is associated with neuroimmune functions and behavioral alterations in ASD.

In another study Ansari et al. investigated if the A2AR signaling pathway regulates cytokine production in *BTBR* mouse (Ansari et al. 2017a), which was previously shown to exhibit elevated proinflammatory cytokines that modulate their behavioral response (Careaga et al. 2015). They examined if A2AR agonists and antagonists affect the levels of IL-2, IL-6, IL-9, interferon gamma (IFN-γ), tumor necrosis factor alpha (TNF-α), and transforming growth factor β (TGF-β) in spleen and in splenic CD4 T lymphocyte cells (CD4 T cells). mRNA and protein levels of these cytokines were also analyzed in the brain tissues. The results showed that SCH 58261, an A2AR antagonist, upregulated levels of pro-inflammatory cytokines, while CGS 21680, an A2AR agonist, restored the balance between pro- and anti-inflammatory cytokines in the *BTBR* mouse, indicating the potential therapeutic promise of CGS 21680 for the treatment of autistic disorders.

#### Limitations

Although these studies show that A2AR regulates the expression of pro and anti-inflammatory cytokines, they do not necessarily prove that these cytokine changes drive immunologic dysfunction or behavioral phenotypes. Therefore, translating these findings into human ASD will require more work.

### C58 Mouse Model (1 Study)

The *C58* inbred mouse strain is an attractive model for repetitive behavior characteristic of neurodevelopmental disorders including ASD. Compared to C57BL/6 mouse, C58 mouse shows high rates of spontaneous hindlimb jumping and backward somersaulting. This makes the model useful for studies deciphering the etiology and pathophysiology of aberrant repetitive behaviors associated with ASD (Whitehouse et al. 2017).

A study by Lewis et al. explored the potential of P1-adenosine receptor agonists to normalize repetitive behaviors associated with neurodevelopmental disorders (Lewis et al. 2019). A combination of adenosine A1 (CPA) and A2AR (CGS21680) agonists were administered to C58 mice each day, for 7 days. These agonists are known to alter the firing frequency of dorsal striatal neurons in the indirect pathway of the basal ganglia. Neither agonist alone was effective; however, their combination significantly reduced repetitive behaviors in both male and female mice over a six-hour period in the 7-day treatment course, indicating a long-lasting effect. Agonist treatment increased the expression of c-Fos, a marker for neuronal activity, in the dorsal striatum, suggesting enhanced activation of the direct and indirect pathways of the basal ganglia, which might have restored the balance in neural activity. The above findings point to P1-purinergic adenosine receptors of the dorsal striatum as prospective therapeutic target for ASD-related repetitive behaviors.

#### Limitations

The high levels of jumping/backward somersaulting behaviors in this model do not fully recapitulate the complexity of ASD-related human repetitive behaviors. Though the agonists here showed behavioral improvement, the study does not support a mechanistic link that drug combination restored indirect-pathway activation. Also, the longer‐term effects of treatment, its potential side‐effects and detailed sex analysis need to be done.

### P2X4R-deficient Mouse Model (1 Study)

P2X4R is a member of the P2X superfamily of ion channels. They are classified as ligand-gated ion channels since their opening is triggered by the binding of a ligand, and are typically expressed by neurons of the brain, microglia, some epithelial and endothelial cells and intracellular compartments like lysosomes. P2X4Rs are the most sensitive of all the P2XRs. They respond to nanomolar concentrations of ATP and play a role in pain signaling, synaptic plasticity and trigger inflammation in response to extracellular (e) ATP (Suurvali et al. 2017). The P2X4-deficient mouse exhibits a plethora of socio-communicative and neurodevelopmental abnormalities akin to other murine models of ASD (Wyatt et al. 2013).

Wyatt et al. investigated the behavioral and neurochemical consequences of P2X4R deficiency in mice (Wyatt et al. 2013). The authors recorded the behavioral responses in 3- to 5-month-old male P2X4R heterozygous (HZ), knockout (KO) and WT mice. Both heterozygous HZ and KO mice displayed reduced social interactions and decreased ultrasonic vocalizations in response to maternal separation, indicating deficits in social communication and sensorimotor deficits Additionally, they showed enhanced tactile sensitivity and significant changes in the expression of glutamate receptor subunits in brain regions such as the prefrontal cortex and hippocampus, including decreased NMDA receptor subunits (GluN2A and GluN2B) and increased AMPA receptor subunits (GluA1 and GluA2). No significant differences were observed in anxiety-related behaviors or locomotor activity between KO, HZ, and WT mice. Collectively these results suggest that P2X4R signaling plays a crucial role in a plethora of socio-communicative and sensorimotor functions that are often affected in ASD, making these receptors an attractive therapeutic target.

#### Limitations

Because the model is a constitutive P2X4 KO, it is difficult to disentangle, if the observed behavioral deficits reflect lifelong adaptations rather than the acute impact of P2X4 on behavior in adulthood or solely on its deletion. Also, only one receptor subtype in one sex, under one set of housing/handling/assay conditions were studied here limiting translation potential. The authors appropriately caution here that mouse social/vocalization assays do not accurately recapitulate human socio-communicative impairments.

### Neuroligin-4 (Nlgn4) KO (Nlgn4-/-) Mouse Model (1 Study)

NLGN4, a postsynaptic cell-adhesion molecule is implicated in human ASD. The encoding gene, *NL4* is mutated in rare cases of familial autism. Its mutation leads to discrete developmental brain disorders, especially in synaptic organization and remodeling (Jamain et al. 2003). Nlgn4 KO (*Nlgn4-/-*) mouse shows increased repetitive behavior and altered social interaction. These conditions are more pronounced in the males, mimicking the higher incidences of ASD in men (El-Kordi et al. 2013).

A study published in 2023, investigated how the loss of Nlgn4 affects microglial cells in the brain, with a focus on sex-specific differences (Guneykaya et al. 2023). Fourteen-week-old *Nlgn4-/-* male mice were treated with 17β-estradiol via drinking water every two days, for 6 weeks. Microglial structure, function and proteomic profile in male and female sexes in WT and *Nlgn4-/-* mice were examined, in the CA3 region of the hippocampus. Male *Nlgn4-/-* mice exhibited reduced microglial density, less complex morphology, and diminished responses to injury in conjunction with alternations in purinergic signaling in the hippocampal CA3 region. The study revealed a sex-specific impairment in P2Y12-purinergic signaling in *Nlgn4-/-* males. Proteomic analysis revealed altered energy metabolism in male microglia, highlighting sexual dimorphism in microglial phenotypes associated with ASD. Furthermore, there was an impairment in male specific gamma oscillations. Gamma oscillations are important for cognition and for modulating the morphology and function of microglia, which are important for synaptic pruning, phagocytosis of pathogens and regulation of inflammation in the brain. Finally, estradiol treatment to male *Nlgn4-/-* mice restored the altered microglial phenotypes and functions, suggesting potential for sex-specific therapeutic strategies.

#### Limitations

Microglia do not express Nlgn. So, the changes reported here do not reflect direct microglial effects and are plausibly secondary to neuronal networks. How the cellular/physiological changes drive behavioral ASD features need further investigation. Importantly this study does not encompass the developmental windows critical for autism and focuses only on the CA3 region of the brain hippocampus. Though the sex-difference findings here are interesting, the underlying cause of female resilience needs further investigation.

### Inositol 1,4,5-triphosphate Receptor Type 2 (IP3R2)-deficient Mouse Model (1 Study)

Astrocytic activation occurs through increased cytoplasmic calcium signals, primarily mediated by IP3R2 (Cao et al. 2013). The IP3R2 gene is affected by rare de novo copy number variants in ASD patients (Gilman et al. 2011).

Wang et al. demonstrated the crucial role of astrocyte-derived ATP in modulating ASD-like behaviors in mice (Wang et al. 2021), using the *IP3R2* null mouse (*IP3R2-/-*), (ii) astrocyte-specific *IP3R2* conditional knockouts (cKOs) which were generated by crossing the Aldh1l1-CreER mice with the IP3R2loxp/loxp mice and (iii) eliminated IP3R2 expression in medial prefrontal cortex (mPFC) astrocytes by injecting adeno-associated virus with *IP3R2*-specific shRNA. Compared to WT, the *IP3R2-/-* mouse showed deficits in social interaction, increased repetitive behaviors and markedly decreased astrocyte somatic Ca^2+^ signals which remained unaffected by GPCR-agonist cocktails. These animals also exhibited enhanced exploratory behaviors and showed no change in anxiety levels or locomotor activity. Similar to the *IP3R2-/-* mice, cKO mice had behavioral abnormalities and Ca^2+^ signaling impairment, but no change in exploratory behaviors or anxiety or locomotion, indicating that IP3R2 deletion is responsible for two core ASD-like behaviors: reduced social interactions and increased repetitive actions. The astrocyte-specific selective deletion by shRNA produced no changes in cognitive impairment or anxiety or locomotion but showed significant alterations in social behavior compared to the *IP3R2-/-* mice. In the astrocytes from *IP3R2-/-* and *IP3R2* cKO mice compared to WT, there was also a drastic reduction in the neurotransmitter ATP, hinting to the role of astrocyte-specific IP3R2. This change was only observed at the level of extracellular (e) ATP, while total ATP and intracellular levels remained unchanged. Reduced eATP release and elevated ecto-ATPase may have contributed to this. The P2Y1R antagonist MRS2500 inhibited ATP release in WT mice, showing P2Y1Rs involvement. Additionally, astrocytes treated with ARL67156 had higher ATP levels in *IP3R2-/-* mice than WT mice, indicating increased ecto-ATPase activity. Injecting ATP before behavior analysis resolved social interaction issues in *IP3R2-/-* animals but not repetitive behaviors. ATP corrected social deficits in *IP3R2* cKO mice without changing social preference. ATPγS infusions in mPFC, improved social impairments in both *IP3R2-/-* and *IP3R2* cKO mice, suggesting mPFC as a potential locus. These effects depended on neuronal P2X2Rs. Knocking down P2X2R in the mPFC, removed the positive effect of ATP and independently induced social deficits in WT mice. P2X2R knockdown also impaired transmission through the GABAA receptors (GABAAR), indicating that astrocyte Ca^2^⁺-induced ATP release enhances cortical GABA inhibition via P2X2Rs. Additionally, the GABAAR agonist clonazepam also rescued social deficits, suggesting impaired GABAergic transmission in the autism-like behaviors. In summary, an astrocyte-derived ATP deficiency led to autism-like behavior in *IP3R2-*deficient mice. Both synaptic and behavioral abnormalities in these mice were partially or completely normalized by ATP application and enhancing GABAergic transmission.

#### Limitations

ATPγS infusion in mPFC did not rescue repetitive behavior, indicating that astrocyte derived ATP via IP₃R2 does not fully explain all ASD-like behaviors in this model. The rescue experiments are acute/short-term manipulations and don’t reflect if chronic modulation over development would change ASD-like phenotype. Furthermore, these investigations were performed in adult astrocytes rather than early/developmental stages crucial for ASD and does not focus on other regions of the brain beyond the mPFC or address sex-specific variations.

### Studies on Environmental Exposures and Purinergic Signaling Dysregulations

#### Valproic Acid (VPA) (5 Studies)

Prenatal exposure to VPA, an environmental risk factor and commonly used drug to treat epilepsy, mood disorders increase the risk of ASD in offsprings (Wood et al. 2015). This association is particularly pronounced when exposure occurs during critical periods of neurodevelopment during pregnancy (Ornoy et al. 2023). Therefore, prenatal exposure to VPA in animals, including rodents, zebrafish have become well-established and widely used preclinical models for studying ASD. These models often exhibit characteristics of idiopathic autism, including deficits in social interaction and communication, increased repetitive behaviors and anxiety with an epigenetic or environmental background (Cohen et al. 2019).

A study published in 2017 investigated how VPA could affect purinergic signaling in adult zebrafish (Zimmermann et al. 2017). The authors reported no change in ATP and ADP hydrolysis or cytosolic adenosine deaminase (ADA) activity. AMP hydrolysis activity increased by 12.5% and ecto-ADA activity decreased by 19.2%. The gene expression levels of the eATP hydrolyzing enzyme, ectonucleoside triphosphate diphosphohydrolase 8 (entpd8), ADA 2.1, and A2a.1 mRNA were increased. These results suggest that embryological exposure to VPA leads to long-term alterations in extracellular nucleotide metabolism, potentially contributing to social behavior deficits in ASD-models.

Babiec et al. also reported that prenatal exposure to VPA affects the expression and activity of purinergic receptors in the developing rat brain (Babiec et al. 2022). Pregnant rats received a single intraperitoneal (i.p.) injection of VPA (450 mg/kg body weight) on embryonic day (E) 12.5, coinciding with neural tube closure. The researchers then analyzed changes in the expression and activity of specific purinergic receptor subtypes in offsprings at embryonic (E) day 19, a critical window in prenatal brain development. Protein levels of adenosine receptors (A1, A2b, A3) and purinergic receptors (P2X2, P2X3, P2X7, P2Y1) changed significantly. These receptors influence progenitor cell proliferation, neuroanatomical changes, synaptic function, neuron migration and differentiation, dendrite and axon formation, as well as glutamate/GABA balance and overall neural excitability (Burnstock 2018). Furthermore, treatment with adenosine, ATP, and the metabolically stable ATP analog ATPƳS significantly increased intracellular Ca^2+^ ([Ca^2+^]i) in cortical neurons from VPA embryos. Stimulation with P2X2/3 receptor agonist α,β-methylene-ATP further elevated [Ca^2+^]i, indicating receptor hyperactivity, while BzATP (P2X7/4R agonist) and ADPβS (P2Y1/P2Y12R agonist) decreased [Ca^2+^]i, pointing to reduced activity. Critically, this study suggests that environmental teratogens like VPA can potentially disrupt purinergic signaling pathways in the developing brain during embryogenesis and thereby contribute to ASD pathogenesis.

A study published in 2020 by Hirsch et al. investigated the effects of a single dose of suramin, a non-selective purinergic antagonist, on behavioral and molecular alterations in VPA-induced rat model of ASD (Hirsch et al. 2020). Pregnant females received a single injection (i.p.) of 600 mg/kg VPA at day E12.5. Male offspring received a single i.p. injection of suramin, 20 mg/kg. Suramin administration restored sociability and reduced anxiety-like behavior. However, it did not improve deficits in reciprocal social interactions or normalize the heightened pain threshold observed in VPA-exposed rats. While suramin did not alter the VPA-induced upregulation of purinergic receptors P2X4 and P2Y2 in the hippocampus or P2X4 in the medial prefrontal cortex, it normalized elevated levels of IL-6, a pro-inflammatory cytokine associated with neuroinflammation in ASD. These results underscore the promise of suramin to address certain ASD-associated behavioral abnormalities.

In 2023 Babiec et al. published a study investigating how prenatal exposure of VPA alters purinergic receptor expression in the brain and affects glial cell morphology in a rat model of ASD (Babiec et al. 2023). Pregnant rats received a single intraperitoneal injection of VPA at embryonic day 12.5 and male offspring were analyzed at postnatal day (P) 52 (adolescence). The hippocampus and cortex were assessed for expression and localization of purinergic receptor mRNA and proteins respectively. The hippocampus of the preclinical ASD rats showed an increase in P2X1R levels, with a decrease in P2X7 and P2Y1Rs, while the cortex had a reduced P2X1R levels. Cortical microglial cell numbers and morphology were significantly altered, and IL-6 levels were elevated. The administration of a non-selective P2R antagonist partially mitigated the glial cell changes. These findings demonstrate that prenatal VPA exposure leads to region-specific alterations in purinergic receptor expression in adolescent brains, contributing to cortical microglial activation/neuroinflammation and underlines the importance of purinergic receptors as potential therapeutic targets for ASD. The administration of a non-selective P2R antagonist partially mitigated the glial cell changes.

​In a study in 2024, Babiec et al. reported that the activation of P2 purinergic receptors led to synaptic alterations, behavioral changes and development of autism-like symptoms in the VPA-injected rat model (Babiec et al. 2024). Wistar female rats were i.p. injected with VPA (450 mg/kg body weight) at E12.5. At P52 the male VPA offspring received a single i.p. injection of the non-selective P2-purinoceptor antagonists PPADS and isoPPADS (12.5 mg/kg body weight). After 24 h. behavioral tests were recorded. Rats were sacrificed the next day after behavioral testings. Reverse phase high performance liquid chromatography (HPLC) analyzed changes in purine metabolism (ATP, ADP, AMP and adenosine) in the cerebrospinal fluid while quantitative real-time polymerase chain reaction (qRT-PCR) and western blots detected mRNA and protein levels in the brain’s hippocampus, cortex and cerebellum. VPA induced hyperactivation of purinergic signaling led to activation of mTOR kinase, the catalytic subunit of the mTORC1 complex responsible for synaptic proteins homeostasis and phosphorylation of downstream eukaryotic translation initiation factor 4E-binding protein 1 (4E-BP1). This led to dissociation of eukaryotic translation initiation factor 4E (eIF-4E), and translation of synaptic proteins such as synaptophysin, vesicle-associated membrane protein ½ (Vamp1/2) and development of autistic behaviors. Inhibition of purinergic P2Rs prevented synaptic and behavioral alterations. This study highlights the role of VPA as an environmental risk factor to alter purinergic signaling in association with induction of ASD.

##### Limitations

VPA-prenatal exposure is only one of many mechanisms associated with ASD and the clinical situation in humans is far more complex, so translational potential remains speculative. Additionally, one dose and/or one time-point of VPA exposure were used in the studies limiting understanding of dose–response, timing effects, and whether different gestational windows would yield different results. Also, causal link between purinergic signaling and behavioral deficits and ASD need to be explored. Suramin’s lack of impact on social interaction deficits and purinergic receptor expression suggests that additional studies are necessary to fully understand its therapeutic potential.

### Maternal Immune Activation (MIA) using Agents like Poly(I:C) and Hyperpurinergia Mouse Model (5 Studies)

The MIA model replicates a potential environmental risk factor for ASD during pregnancy (Ellul et al. 2023). In 1960, after the U.S. rubella epidemic, it was first proposed that there might be a direct link between neurodevelopmental disorders like ASD and infection during pregnancy (Chess 1971). A fair amount of research over the years indicates that MIA during first and second trimesters of pregnancy, rather than infection by itself enhances the risk of ASD in offsprings. The severity of the infection depends on the dose of injection and the timing of exposure during pregnancy (Hornig et al. 2018). Rodent models generated by administration of either double-stranded RNA [polyinosinic:polycytidylic acid poly (I:C), for mimicking viral infection], or lipopolysaccharide (LPS, for mimicking bacterial infection) have helped to elucidate the link between systemic maternal inflammation and MIA-induced neuroinflammation in ASD offsprings. The offsprings typically exhibit behavioral problems, such as reduced social interaction, anxiety and repetitive behaviors seen in ASD (Bao et al. 2022). Hyperpurinergia likewise plays a significant role in the development of ASD-like features. The hyperpurinergia model here investigates the effects of eATP in typical mice and the cumulative impact of hyperpurinergia on prenatal MIA offspring (Zolkipli-Cunningham et al. 2021).

A study by Naviaux et al. in 2013 investigated the effects of suramin on autism-like features in a mouse model(Naviaux et al. 2013). Researchers utilized the MIA mouse model, in which pregnant mice were exposed to poly(I:C) either once (E12.5) or twice (E12.5 and E17.5) to simulate viral infection. The resulting offspring exhibited ASD-like characteristics. Saline injected dams served as controls. Mice were treated with suramin at 6-weeks of age (10 or 20 mg/kg ip) and at 8-weeks animals were subjected to a series of tests. At 16-weeks, the males were sacrificed for analysis of cerebral synaptosomes and mitochondria, hematological tests, immunoglobulins, corticosterone, neuropathy and immunohistochemistry. Suramin treatment improved social preference, sensitometer coordination, metabolic rate/oxygen consumption and cerebral mitochondrial activity while normalizing body temperature. Neurobiological studies showed that this anti-purinergic agent prevented cerebellar Purkinje cell loss and corrected the synaptic structural abnormalities. The expression of purinergic receptors (P2Y2 and P2X7) and levels of Fmrp, which were reduced in the MIA mice, were also restored by suramin. Finally, suramin treatment corrected metabolic dysfunctions, increased cholinergic signaling in cerebral synaptosomes via the receptor nicotinic acetylcholine receptor subunit a7 (nAchRa7) and normalized signaling pathways involving ERK1/2 and CAMKII. This study underlines that aberrant purinergic signaling plays a crucial role in the development of ASD-like features and illustrates how anti-purinergic therapy with suramin can reverse these abnormalities in a mouse model. These findings provide a basis for further research into purinergic signaling as a potential therapeutic target for ASD.

In the following year, Naviaux et al. investigated the efficacy of a single dose of suramin to ameliorate the behavioral and metabolic abnormalities in the MIA-mouse model of autism (Naviaux et al. 2014). Male mice born to MIA-pregnant dams [administered two i.p. injections of Poly(I:C)] were treated with a single dose (20 mg/kg, i.p.) of suramin at 5.25-months of age, which is the biological equivalent of 30 years in humans. Two-days post-treatment, animals were evaluated for behavioral tests for novelty preference, social interaction, sensorimotor coordination, anxiety and light-avoidance. Five weeks after the suramin dose, half of the animals were sacrificed and their tissue levels of suramin were quantitated. The other half was again injected with the suramin at 7.5 weeks and sacrificed two days later to detect acute tissue levels of the suramin. Additionally, untargeted metabolic profiling was performed by mass spectrometry to determine the plasma metabolite levels of the animals that were screened for behavioral analysis. The effects of suramin peaked on day 2 and largely faded after 5 days, coinciding with its half-life. Metabolic pathways that were normalized were purine metabolism (ATP, ADP, AMP and adenosine levels) a key cell signaling pathway hypothesized to drive ASD-like symptoms, lipid metabolism which is crucial for brain signaling, and redox metabolism (balance of NADH/NAD +, glutathione systems) frequently associated with autism related oxidative stress, mitochondrial metabolism also known to be affected in autism, amino acid metabolism (glutamate, GABA) crucial for neurotransmission and excitatory and inhibitory balance, tryptophan metabolism which regulates mood and behavioral changes in ASD and others including glucose, nucleotide and carbon metabolism. Notably, two doses of suramin given at 6.5 and 6.75 months of age did not improve performance. This important study sheds light on a new mechanism wherein a drug like suramin that does not cross the blood–brain-behavior still affects the central nervous system to correct the behavioral and metabolic deficits in a MIA-mouse model of ASD in only a single dose.

Later in 2021, Zolkipli-Cunningham et al. examined the metabolic disturbances and behavioral anomalies associated with ASD (Zolkipli-Cunningham et al. 2021). This study evaluated the effects of eATP (representing acute hyperpurinergia) in a typically developing mouse model and assessed if eATP injections could influence MIA mice. For hyperpurinergia, mice were administered intraperitoneal (i.p.) doses of nucleotides (20 μl/g. doses up to 0.5 μmol/g) and intravenous (i.v.) doses (5 μl/g. doses up to 0.5 μmol/g) by lateral tail vein. MIA was initiated in pregnant dams by i.p. injection of poly (I:C); 2 mg/kg i.p.) on day E12.5 and E17.5. Postnatal MIA challenge [poly(I:C); 2 mg/kg i.p.] was given to 8–9-month-old animals. The corresponding controls received normal saline. The study revealed that systemic eATP injections cause significant metabolic disturbances leading to (i) Bioenergetic disruptions: There was a 74% decrease in basal metabolic rate, as measured by oxygen utilization (VO₂), pointing to reduced oxygen consumption and hypothermia in males and females with sex-specific sensitivities for the different purine metabolites. (ii) Behavioral abnormalities: Male mice showed increased sensitivity to behavioral tests such as reduced activity and increased anxiety-like behaviors. The nature and magnitude of response to eATP injection also varied between the males and females. Likewise, MIA mice injected postnatally with eATP, or poly (I:C) showed (i) Exacerbation of the behavioral abnormalities (ii) Changes in their metabolic profiles, indicating a compounded effect of prenatal immune activation and postnatal hyperpurinergia. In summary, the study suggested that purinergic signaling is involved in ASD pathophysiology and modulating purinergic pathways could provide new therapeutic options, particularly for cases involving prenatal immune dysregulation. Furthermore, understanding sex-based differences in hyperpurinergic responses may lead to personalized treatments.

The above three preclinical studies add strong evidence to the role of prenatal immune activation in ASD and shed light on how hyperpurinergia may complicate it. They also explore the therapeutic promise of suramin, a nonspecific P2YR and P2XR antagonist in ASD.

A study published by Horva´th et al. in 2019 examined the role of P2X7Rs in mediating autism-like behaviors in mice following poly(I:C)-induced MIA (Horvath et al. 2019). This is the first report on the role of P2X7R’s in MIA-induced autism. Offsprings of WT *P2X7*R mice injected with poly(I:C) at E12.5 (3 mg/kg i.p.) and E17.5 (1.5 mg/kg i.p.) were examined between P60─P90. Mice displayed decreased social interaction, lack of motor coordination and increased repetitive behavior. Such behavioral alterations were not observed in the poly(I:C)-treated *P2X7*R-/- offsprings or in the vehicle-injected controls. The cerebellar lobe VII of fetal-brains from MIA-induced WT *P2X7*R dams had lower number of Purkinje neurons, more malformed synaptosomes and increased IL-6 and ATP and decreased AMP levels compared to the corresponding *P2X7*Rs-/- mice. The maternal plasma collected 2 h after a single i.p. injection of poly(I:C) [3 mg/kg] had significantly elevated IL-6, IL-1α and KC. Cortical development was also disrupted in the WT but not in the *P2X7*R-/- mice subjected to MIA as revealed by immunostaining for T-brain1, a marker for neural migration/axon guidance. Importantly, a single injection of the selective P2X7R antagonist (JNJ47965567, 30 mg/kg i.p.) to pregnant WT dams 2 h before poly (I:C) administration prevented the development of ASD-like behaviors and the morphological abnormalities like Purkinje cell dropout and malformed synaptosomes in their offsprings. Both genetic deletion and pharmacological inhibition of P2X7Rs in mothers by antagonist JNJ47965567 led to reduced levels of IL-6 in maternal plasma and decreased cytokine levels in fetal brains, indicating that maternal P2X7R is essential to modulate inflammatory responses associated with MIA in offspring. Inhibiting P2X7Rs after birth alleviated behavioral and morphological abnormalities, suggesting that P2X7R activity continues to influence neurodevelopment beyond the prenatal period. These effects were attenuated in the *P2X7*Rs-/-. Administration of the P2X7R agonist (ATP, 400 µM) also resulted in ASD like behavioral and morphological changes observed with MIA challenge which were also attenuated in the *P2X7*-/- mice, confirming the role of P2X7Rs in the ATP-induced autistic alterations in WT offsprings. Together, this study provides evidence that P2X7R activation is a pivotal mechanism by which MIA leads to autism-like behaviors in offsprings. These findings open potential therapeutic strategies targeting P2X7Rs to prevent or mitigate neurodevelopmental pathologies associated with maternal immune challenges and hyperpurinergia, an overactive state of purinergic signaling.

In a related study in 2022, Szabó et al. tested whether blocking P2X7 signaling or its downstream NLRP3/IL-1β pathway during pregnancy would prevent ASD-like phenotypes in offsprings (Szabo et al. 2022). Pregnant dams (WT, *P2X7*R-/- or IL-1α/β-/-) were injected (i.p.) with the viral mimetic Poly(I:C) at E12.5 (3 mg/kg) and E17.5 (1.5 mg/kg). To evaluate the pharmacological interventions, pregnant females were treated 2 h before poly(I:C) challenge with either MCC950 (50 mg/kg), a selective NOD-LRR-and pyrin domain-containing protein 3 (NLRP3) inflammasome inhibitor or with an anti-IL-1β neutralizing antibody or with the selective P2X7R antagonist, JNJ47965567 (30 mg/kg). For postnatal challenge, males received daily injections of JNJ47965567 (20 mg/kg) for nine days, starting at P25. Vehicle (saline or captisol) injected mice served as controls. Between (8–10 weeks age) the male offspring were analyzed for behavioral deficits, neuroanatomical changes and were subjected to cytokine profiling. Parallel groups of WT or *P2X7R*-/- dams (with or without JNJ 47965567/MCC950) were used to determine how P2X7/NLRP3 influenced these inflammatory signals. Consistent with the previous study from this group, male pups from WT MIA-dams had behavioral abnormalities reflective of ASD. Neither *P2X7R*-/- offsprings nor saline injected vehicular controls developed these social deficits or repetitive behaviors after MIA challenge. Maternal MCC950 pretreatment abolished the behavioral deficits seen with MIA. Similarly, maternal injection of a neutralizing IL-1β antibody prevented both the social and repetitive deficits in offspring. Therefore, blocking NLRP3 or IL-1β during pregnancy abolished the development of autistic characteristics in offspring. IL-1α/β double-knockout dams (unable to signal through IL-1) likewise yielded offspring resistant to MIA; IL-1-deficient offspring showed no ASD-like behaviors after poly(I:C).​ Blocking P2X7 signaling in offspring with daily injections of JNJ47965567, rescued the behavioral anomalies that developed after a maternal insult. Poly(I:C) injection significantly elevated maternal IL-1β, IL-6, IL-2, and chemokine monocyte chemotactic protein 1 (MCP-1) levels in the placenta and fetal brains. Genetic deletion or pharmacological blockade of P2X7R abrogated this effect, indicating the role of P2X7Rs. Likewise, maternal MCC950 completely prevented poly(I:C)-induced IL-1β accumulation in placenta/fetal brains and the development of neuroanatomical alterations like synaptosome deformities and Purkinje cell loss. *P2X7R-/-* offspring likewise showed no neuroanatomical alterations after MIA​. Together, these results confirm that maternal P2X7/NLRP3 activation is necessary to trigger MIA-induced inflammatory cascades and brain deformities and provides strong evidence that the P2X7–NLRP3–IL-1β pathway is a key link between maternal immune activation and autism like traits in mouse. These findings suggest that biomarkers of maternal inflammation (e.g. IL-1β, and chemokines RANTES and MCP-1) may predict ASD risk and demonstrate the promise of the P2X7 antagonist, JNJ47965567 to reduce ASD-like behaviors in preclinical studies. It also opens avenues where P2X7 blockers or IL-1β antagonists can be used in high-risk pregnancies to prevent ASD-risks.

#### Limitations

The MIA induction timings used in these studies may model a subset of developmental windows; whether this maps precisely to human prenatal immune activation windows or captures all relevant neurodevelopmental cascades is unclear. Furthermore, the MIA model represented here does not fully capture the complexities of ASD in the human population. Additionally, suramin being a non-specific drug can have potential side effects on prolonged chronic use.

### Maternal Gestation-onset Vitamin A Deficiency (VAD) Rat Model (1 study)

Vit A is crucial for neural differentiation and neurite outgrowth (Janesick et al. 2015). Its deficiency was reported in autistic children (Cheng et al. 2021). The maternal gestation-onset VAD rat model has significantly lower Vit A levels (lower than 0.70 μmol/L) than the corresponding controls.

In 2023, a study by Cheng et al. investigated the impact of maternal vitamin (Vit) A deficiency on offspring development (Cheng et al. 2023). Three-week-old female rats fed with Vit A-normal (VAN, 6500 IU/kg VA) and Vit A deficient (VAD, 400 IU/kg VA) diets, were mated with same group VAN or VAD males. Offsprings (P43) born to VAN and VAD mothers, were subjected to behavioral assessments, while gastrointestinal (GI) function was evaluated through measures VAD offsprings exhibited ASD-like behaviors including deficits in social interaction, lack of social novelty, increased grooming and impaired GI function, like motility disorder leading to constipation. To understand if metabolic abnormalities accompanied the behavioral alterations and GI motility issues, the researchers conducted untargeted metabolomics analyses by LC–MS/MS from prefrontal cortex (PFC) and fecal samples from VAN and VAD offspring rats. There was significant alteration in metabolism in brain and GI tract between the two groups, especially in purine and tryptophan metabolism. Inosine (an anti-inflammatory purine metabolite), guanine, inosine monophosphate (IMP) and guanosine monophosphate (GMP) were significantly downregulated in the PFC of VAD offsprings, while uric acid (UA), a pro-inflammatory purine metabolite, critical for cognition, increased in the VAD offsprings. Their fecal samples likewise showed higher levels of adenosine, crucial for neurodevelopment and microglial and astrocytic functions. Furthermore, PFC of VAD offspring rats showed significant dysregulation in tryptophan, tyrosine and phenylalanine biosynthesis pathways. The tryptophan metabolism pathway (tryptophan and 2-aminobenzoic acid increased, 6-hydroxymelatonin and L-kynurenine decreased) was also altered in VAD feces. Together, these disruptions in purine and tryptophan metabolism might have significantly contributed to the behavioral and neurodevelopmental abnormalities of the VAD offsprings, suggesting that maternal VAD may contribute to the development of ASD-like behaviors and GI dysfunction in offspring by disrupting specific metabolic pathways. The study underscores the importance of studies for vit A intake during pregnancy for neurodevelopmental and gastrointestinal health.

#### Limitations

The severity and deficiency of vit A and overall malnutrition in humans needs in-depth investigation before translating these studies. Further the links between altered purine and tryptophan metabolism and observed behaviors remain associative than causal. The limited sample sizes and absence of gender-based studies further restrict the interpretation and practical use of these findings.

### Potential Mechanisms Linking Altered Purinergic Signaling to Autistic Features

#### Impact on Synaptic Function and Plasticity

Prolonged activation of P2Y2/P2Y6R in FXS cortical astrocytes via ATP and UDP elevated [Ca^2+^]i responses and modulated cortical synaptic transmission (Reynolds et al. 2021b). Similarly, elevated levels of ATP in VPA models led to overactivation of neural and glial P2XRs, potentially contributing to synaptic hyperexcitability, a feature often implicated in ASD (Babiec et al. 2024). Overstimulation of P2Y1R in the cortex of VPA rats affected their cognitive abilities (Babiec et al. 2023). Conversely downregulation of P2Y2 and P2X7Rs in the cerebral synaptosomes of the MIA model, reflect disruption of normal synaptic development and plasticity, as these receptors are critical for these processes (Naviaux et al. 2013). Inhibiting the P2Rs in the VPA model prevented the modifications in the synaptic proteins, a feature implicated in ASD (Babiec et al. 2022, 2023). Further deletion of *IP3R2* in astrocytes leads to synaptic deficits (Wang et al. 2021)*.* This finding further supports the hypothesis that dysregulated purinergic signaling affects synaptic dysfunction and leads to behavioral deficits in ASD.

### Role in Neuroinflammatory Processes and Glial Activation

FXS cortical purinergic signaling activated the TNC-TLR4-STAT3-IL6 pathway, leading to IL-6 release and indicating purinergic regulation of pro-inflammatory pathways (Reynolds et al. 2021a). In the BTBR ASD-mouse model, dysregulation in the A2AR purinergic signaling led to alteration in neuroimmune functions through the Th17/RORt pathway, which changed their behavior (Ansari et al. 2017b). A2AR signaling also regulated the pro-inflammatory responses in BTBR whole mouse brains, important for their behavioral responses (Ansari et al. 2017a). Overactivation of the microglial P2X7Rs in the cortex of VPA rat model triggered the release of pro-inflammatory cytokines, leading to neuroinflammation (Babiec et al. 2023). Conversely the downregulation of P2X7Rs in the hippocampus of VPA rats was implicated in microglial activation in that specific brain region (Babiec et al. 2023). Increased expression of P2Y12Rs in the cortex of VPA rats is also associated with microglial activation and inflammation (Babiec et al. 2023). In the MIA mouse model, anti-purinergic therapy corrected the downregulation of P2Y2 and P2X7Rs (in the cerebellum) and normalized the phosphorylation of downstream signaling molecules, further supporting a link between hyperpurinergia and neuroinflammatory processes in this model (Naviaux et al. 2013). Maternal P2X7/NLRP3 activation triggers MIA-induced inflammatory cascades in the whole fetal brains and cell deformities in the cerebellar Purkinje cells leading to autism (Szabo et al. 2022).

### Influence on Neuronal Excitability and Network Activity

In the C58 mouse model of ASD, aberrant adenosine A1 and A2R signaling altered the firing frequency/activity of the neurons in the brain’s dorsal striatum—a major input to the basal ganglia. This concerted activity contributed to repetitive behavioral patterns in these animals (Lewis et al. 2019). Interestingly impairment of P2Y12-purinergic signaling in *Nlgn4-/-* males reduced their neural network activity (in the hippocampal CA3 region), impacted their hippocampal gamma oscillations, synaptic and microglial functions, contributing to neural inflammation and ASD progression in a sex-specific manner (Guneykaya et al. 2023). Furthermore, increased levels of P2X1Rs in the hippocampus of VPA rats, elevated hippocampal long-term potentiation (LTP) by stimulating the release of glutamate, a primary excitatory neurotransmitter. This might have altered the neuronal excitability within the hippocampus (Babiec et al. 2023). Conversely, reduction in P2X1R levels in the cortex of VPA rats disrupted normal excitatory synaptic transmission (Babiec et al. 2023). Dysregulation of CD39 ectonucleotidases and adenosine-based purine metabolites led to synaptic and network dysfunctions in *Fmr1-/-* cortical astrocytes (Reynolds et al. 2024b). These findings suggest that altered purinergic signaling could directly impact neuronal excitability through receptors and indirectly through the neuromodulatory effects of adenosine.

### Potential Interactions with Other Signaling Pathways Implicated in ASD

Preclinical studies have shed light on the potential interactions between purinergic signaling and other signaling pathways, implicated in the pathophysiology of ASD. For example, in the *Fmr1-/-* mouse model, absence of Fmrp increased interactions between A2AR and mGlut5Rs within the hippocampal slices. This also boosted mTOR/mGlu5R, TrkB/BDNF, and STEP signaling pathways which led to synaptic changes, learning and memory deficits (Ferrante et al. 2021). Similarly, in the VPA model, prenatal overstimulation of purinergic receptors was linked to increased mTOR kinase activity in the hippocampus, cortex and cerebellum. This is associated with ASD (Babiec et al. 2024). Blocking P2 receptors in this model prevented mTOR overactivation (Babiec et al. 2024). Likewise, P1 purinergic A2ARs and retinoid-related orphan receptor gamma t (RORγt)/Th17’s regulates neuroimmune functions in the brain and spleen tissues, often dysregulated in autism (Ansari et al. 2017b). Dysregulated purine and tryptophan metabolism in the PFC and gut were shown to contribute to co-existing behavioral and GI symptoms in vit A deficient autism (Cheng et al. 2023). These findings suggest a complex interplay between purinergic signaling and other ASD-related pathways, indicating that the dysregulation of purinergic signaling might exacerbate the abnormalities in the other critical pathways.

### Integrated Perspective on the Role of Purinergic Signaling in Preclinical Models in ASD

**Fig. 5:**
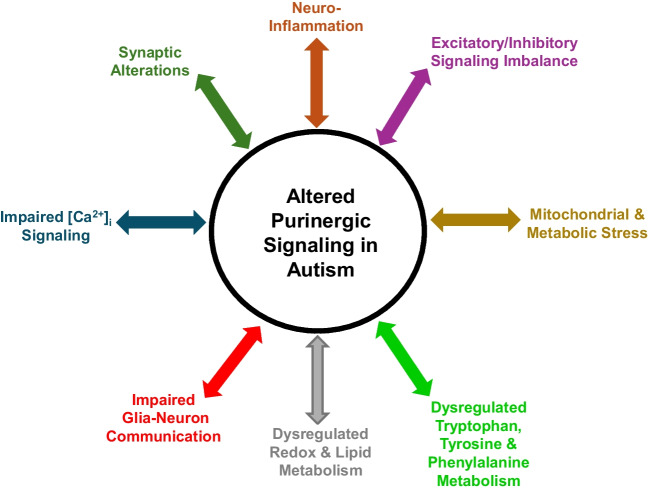
Mechanisms linking altered purinergic signaling to autism

**Fig. 6:**
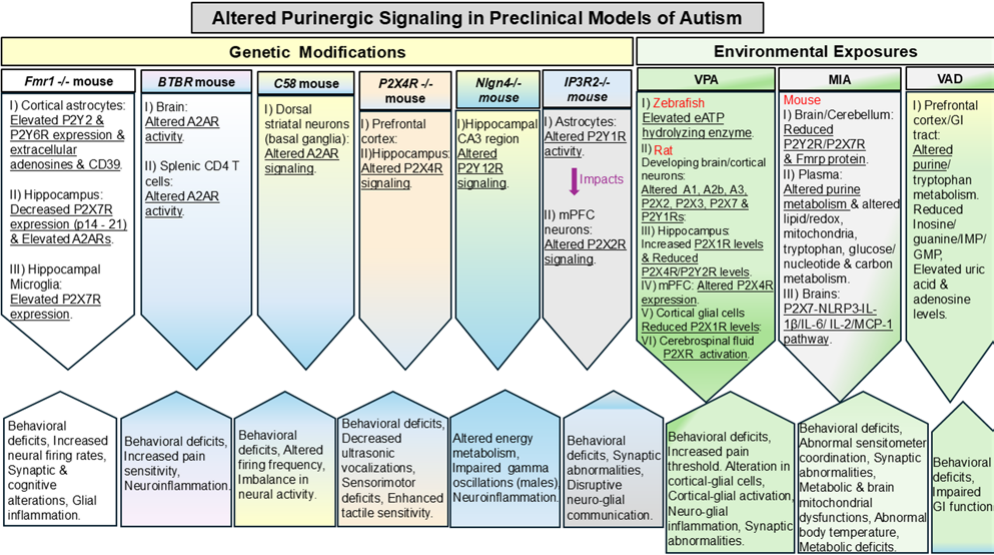
Overview of altered purinergic signaling pathways in preclinical models of autism reflecting the link between genetic modifications, environmental susceptibilities and alterations in the downstream cellular targets/pathways that contribute to its pathophysiology. The convergent patterns across the models involving ATP/adenosine metabolism, P1/P2 receptor alterations, and downstream signaling pathways associated with autism are depicted here

This review indicates that purinergic pathways may regulate broader ASD-related biological networks. The dysregulation of P2X7, P2Y2, A2ARs and ectonucleotidases play a major role in linking purinergic changes to microglial activation, glutamatergic imbalance, as well as neuro-modulatory dysfunction. The findings collectively emphasize the importance of exploring purinergic signaling not only as an isolated pathway but as a dynamic contributor within broader neurobiological networks implicated in ASD (Fig. 5). It, therefore, represents a crucial mechanistic link between genetic susceptibility, environmental exposures, and the down-stream cellular changes that underpin ASD-related neurodevelopmental and behavioral phenotypes (Fig. 6). By altering diverse processes such as synaptic transmission, neuroinflammation, balance of neurotransmitters/neural excitability, glia-neural communication, redox/lipid and protein metabolism, [Ca^2+^]_i_ signaling and mitochondrial metabolism, dysregulated purinergic pathways can exacerbate or even initiate pathological events leading to autism-like traits. This integrated understanding highlights the importance of further dissecting purinergic mechanisms in preclinical models to identify new molecular targets and modify strategies.

### Therapeutic Potential of Targeting Purinergic Signaling in Preclinical Studies

#### Studies Using Purinergic Receptor Agonists or Antagonists

Multiple studies have shown that purinergic receptor antagonists can be therapeutically effective in preclinical models of ASD. For example, AR–C 118925XX, a selective P2Y2R antagonist, normalized the enhanced firing rate of cortical neurons in *Fmr1-/-* neuron-astrocyte co-cultures (Reynolds et al. 2024a). Additionally, inhibiting non-selective P2 receptors with antagonists such as PPADS and isoPPADS in the microglial cells of PFC led to the reversal of autism-like behaviors in the VPA rat model. These antagonists also prevented alterations in the expression of cortical synaptic proteins and the overactivation of mTOR kinase observed in these animals (Babiec et al. 2024). Blocking purinergic A2ARs with istradefylline, restored hippocampal synaptic and cognitive alterations in *Fmr1-/-* mice (Ferrante et al. 2021). In BTBR mouse, activation of A2ARs with CGS 21680 reduced inflammation while inhibitor SCH 5826 reversed this (Ansari et al. 2017b). Furthermore, estradiol treatment to male *Nlgn4-/-* mice rescued their microglial functions in the hippocampal CA3 region (Guneykaya et al. 2023) while the GABAAR agonist clonazepam rescued social behavioral deficits in the *IP3R2-*deficient mice by restoring cortical GABAergic transmission (Wang et al. 2021). Vit A intake improved autism like-behavioral alterations and GI motility issues in VAD rat models (Cheng et al. 2023).

Similarly, anti-purinergic therapy with suramin, another non-selective antagonist for P2Y and P2X7R, normalized the expression of the Fmrp and several purinergic receptors in the MIA mouse model. Suramin also restored neuroanatomical abnormalities and corrected the social deficits, sensorimotor coordination abnormalities, and hypothermia in this model (Naviaux et al. 2014, 2013). Furthermore, a single dose of suramin in VPA-induced rat model of ASD restored sociability, decreased anxiety levels, and normalized the elevated levels of the pro-inflammatory cytokine IL-6 in the medial PFC (Hirsch et al. 2020). These consistent findings across different animal models that block purinergic receptors and alleviate several autism-like behaviors strongly suggest that hyperpurinergia plays a significant role in the pathophysiology of ASD. This highlights the considerable therapeutic potential of targeting this signaling pathway for the development of novel treatments for ASD. The therapeutic actions of suramin might be through its ability to influence mitochondrial function and modulate immune reaction, both of which are implicated in the pathogenesis of ASD (Castora 2019; Szabo et al. 2022).Given its broad range of effects on autism-like behaviors and the underlying biological abnormalities in preclinical models, suramin represents a promising lead compound for the development of more targeted therapies. However, it is important to note that suramin is a non-selective agent and may have off-target effects, making it difficult to determine which specific receptors or downstream signaling pathways are responsible for the observed therapeutic effects. Therefore, understanding the precise mechanisms of action of suramin and its effects on various aspects of ASD pathology in animal models is crucial for guiding future efforts in therapeutic development.

Interestingly, a single injection of the selective P2X7R antagonist JNJ47965567 to pregnant dams prior to the administration of a viral mimetic, prevented ASD-like behaviors and morphological abnormalities in the fetal brains and abrogated their pro-inflammatory responses. Furthermore, even after a maternal immune insult, daily injections of JNJ47965567 to the offsprings reduced their social deficits and repetitive behaviors, thereby underlining the promise of a selective P2X7R antagonist in mitigating ASD-related neurodevelopmental anomalies after birth (Horvath et al. 2019; Szabo et al. 2022).

Together this necessitates the development of more specific purinergic modulators for potential clinical use. Such selective tools will advance our understanding of the intricate roles of different purinergic receptors in the neuropathology of ASD and guide the development of future therapies.

### Potential for Developing Novel Therapies for ASD

The complexity of the purinergic signaling system offers a multitude of potential therapeutic targets for ASD. One promising avenue involves the development of more selective agonists or antagonists like JNJ47965567, that target specific receptors and their subtypes and selectively modulate their activity to correct the errors in the ASD models. This has promise as it might be possible to achieve more precise therapeutic interventions with fewer off-target effects compared to non-selective drugs like suramin. Another potential strategy could be to modulate the activity of ectonucleotidases, such as CD39 and CD73, to restore the physiological balance between ATP and adenosine signaling in the extracellular space. Additionally, Vit A supplementation during pregnancy holds promise in alleviating GI related symptoms in autism (Cheng et al. 2023). The identification of the specific components of the purinergic signaling pathway that are most consistently and critically altered in preclinical models of ASD will be crucial for guiding the development of these novel and more targeted therapeutic strategies.

## Conclusions

This systematic review underscores the pivotal role of purinergic signaling in the pathophysiology of ASD. The animal models addressed here are broadly categorized into (a) genetics, which involve mutations in specific genes linked to ASD in humans, and (b) environmental, which examine the effects of prenatal or postnatal exposure to environmental risk factors on neurodevelopment. Given that interactions between genetic factors and environmental toxins contribute to the development of autism (Pugsley et al. 2022), diverse models provide researchers with valuable opportunities to examine purinergic signaling across specific etiological subtypes of autism.

Analysis of 23 preclinical studies revealed that dysregulation of purinergic pathways contributes significantly to neuroinflammation (Babiec et al. 2023; Naviaux et al. 2013; Reynolds et al. 2021a), persistent synaptic abnormalities (Babiec et al. 2024; Reynolds et al. 2021b), disrupted glial communication (Babiec et al. 2023), and behavioral changes associated with autism-like traits (Hirsch et al. 2020; Horvath et al. 2019; Naviaux et al. 2013). Alterations were detected across numerous components of the purinergic system, including nucleotide and nucleoside levels (Babiec et al. 2024; Zolkipli-Cunningham et al. 2021), receptor expression and function (Babiec et al. 2023; Hirsch et al. 2020; Napier et al. 2024), as well as enzymes important for nucleotide metabolism (Reynolds et al. 2024b; Wang et al. 2021; Zimmermann et al. 2017).

Genetic and environmental factors contribute to mitochondrial, oxidative, metabolic, or immune stress in neural and glial cells within the mPFC, hippocampus, cortex, and cerebellum during brain development (Babiec et al. 2022, 2023; Ferrante et al. 2021; Wang et al. 2021). They affect the GI tract(Cheng et al. 2023). Such stress leads to cell death and the release of extracellular purines (ATP, ADP, UTP, UDP), which act as death signals (Babiec et al. 2024; Reynolds et al. 2024b; Zolkipli-Cunningham et al. 2021). Elevated ATP levels activate the P2X7-NLRP3 inflammasome, triggering the release of pro-inflammatory cytokines that affect disease progression (Szabo et al. 2022). Chronic ADP-mediated P2YR activation leads to atypical excitatory synaptic responses in cortical astrocytes (Reynolds et al. 2021b) and increases IL-6 transcription (Reynolds et al. 2021a). Extracellular ADP and UTP elevates [Ca^2+^]_i_ level in these cells (Reynolds et al. 2021b). Additionally, A2AR-dependent Tc-TrkB pathways alter BDNF and STEP signaling in the cortex and hippocampus, that can subsequently impact synaptic function and cognition (Ferrante et al. 2021). Astrocytic P2X2R knockdown diminishes Ca^2^⁺-induced ATP release, affecting cortical GABAergic transmission and results in altered behaviors observed in autism models (Wang et al. 2021). Prenatal toxin exposure (such as VPA) further increases extracellular nucleoside levels in the brain, glial cells, and CSF, and changes purinergic receptor expression in multiple regions of the adolescent brain (Babiec et al. 2022, 2023; Hirsch et al. 2020). MIA induces pro-inflammatory cascades and brain deformities leading to ASD (Naviaux et al. 2014, 2013; Zolkipli-Cunningham et al. 2021) while maternal Vit A deficiency disrupts purine and tryptophan metabolism in offsprings and contributes to the development of ASD-like behaviors (Cheng et al. 2023).

To elucidate these complex mechanisms, a multidisciplinary approach integrating molecular, behavioral, and pharmacological analyses is essential. Given the multifactorial nature of autism, no single animal model can fully reproduce the complexity and heterogeneity observed in human ASD (Li et al. 2021). Results from one model may not accurately reflect the range of genetic or environmental causes associated with ASD. Comparative analysis across diverse models will facilitate identification of both shared and model-specific mechanisms by which purinergic signaling may impact this disorder. Furthermore, experimental conditions often vary across studies, including differences in the age of animals used, dosage and timing of drug administration, and methods for assessing autism-like behaviors (Li et al. 2021). Such variability can contribute to inconsistencies in findings. It is important to consider both the specific model and experimental design when interpreting these results. Nevertheless, consistent findings across animal models reinforce the hypothesis that the purinergic system constitutes an attractive target for innovative therapeutic strategies.

Future research should focus on identifying receptor subtypes crucial to specific disease phenotypes, clarifying the temporal dynamics of purinergic dysregulation and optimal intervention periods, validating the translatability of animal research to clinical populations, and exploring gender differences related to purinergic signaling in ASD. Furthermore, functional studies investigating mechanistic links between purinergic dysregulation and other core ASD mechanisms will be critical for uncovering druggable targets and elucidating underlying molecular pathways. Assessment of alternative, safer, and more selective therapeutic agents—beyond suramin, using patient-derived in vitro models may support development of personalized interventions. Investigating long-term outcomes of purinergic modulation on neurodevelopmental trajectories, neuro-glial health, and synaptic plasticity—in addition to short-term behavioral improvements—is also imperative.

Concluding, this review highlights purinergic signaling as a promising yet underexplored area within autism research. Targeted modulation of these pathways offers considerable potential for the development of novel treatments to ameliorate ASD symptoms.

## Data Availability

No datasets were generated or analysed during the current study.

## References

[CR1] Ansari MA, Attia SM, Nadeem A, Bakheet SA, Raish M, Khan TH, Al-Shabanah OA, Ahmad SF (2017a) Activation of adenosine A2A receptor signaling regulates the expression of cytokines associated with immunologic dysfunction in BTBR T(+) Itpr3(tf)/J mice. Mol Cell Neurosci 82:76–87 28465254 10.1016/j.mcn.2017.04.012

[CR2] Ansari MA, Nadeem A, Attia SM, Bakheet SA, Raish M, Ahmad SF (2017b) Adenosine A2A receptor modulates neuroimmune function through Th17/retinoid-related orphan receptor gamma t (RORgammat) signaling in a BTBR T(+) Itpr3(tf)/J mouse model of autism. Cell Signal 36:14–24 28438638 10.1016/j.cellsig.2017.04.014

[CR3] Antonioli L, Pacher P, Vizi ES, Hasko G (2013) CD39 and CD73 in immunity and inflammation. Trends Mol Med 19:355–367 23601906 10.1016/j.molmed.2013.03.005 PMC3674206

[CR4] Babiec L, Wilkaniec A, Adamczyk A (2022) Prenatal exposure to valproic acid induces alterations in the expression and activity of purinergic receptors in the embryonic rat brain. Folia Neuropathol 60:390–402 36734381 10.5114/fn.2022.123999

[CR5] Babiec L, Wilkaniec A, Matuszewska M, Palasz E, Cieslik M, Adamczyk A (2023) Alterations of purinergic receptors levels and their involvement in the glial cell morphology in a pre-clinical model of autism spectrum disorders. Brain Sci 13(7):1088 37509018 10.3390/brainsci13071088 PMC10377192

[CR6] Babiec L, Wilkaniec A, Matuszewska M, Palasz E, Cieslik M, Adamczyk A (2024) Correction: Babiec et al. alterations of purinergic receptors levels and their involvement in the glial cell morphology in a pre-clinical model of autism spectrum disorders. Brain Sci 14(3):233 37509018 10.3390/brainsci13071088 PMC10377192

[CR7] Bakker CE, Oostra BA (2003) Understanding fragile X syndrome: insights from animal models. Cytogenet Genome Res 100:111–123 14526171 10.1159/000072845

[CR8] Bao M, Hofsink N, Plosch T (2022) LPS versus poly I:C model: comparison of long-term effects of bacterial and viral maternal immune activation on the offspring. Am J Physiol Regul Integr Comp Physiol 322:R99–R111 34874190 10.1152/ajpregu.00087.2021 PMC8782664

[CR9] Bernardet M, Crusio WE (2006) Fmr1 KO mice as a possible model of autistic features. Sci World J 6:1164–1176 10.1100/tsw.2006.220 PMC5917219 16998604

[CR10] Burnstock G (1972) Purinergic nerves. Pharmacol Rev 24:509–581 4404211

[CR11] Burnstock G (2007) Purine and pyrimidine receptors. Cell Mol Life Sci 64:1471–1483 17375261 10.1007/s00018-007-6497-0 PMC11149472

[CR12] Burnstock G (2018) Purine and purinergic receptors. Brain Neurosci Adv 2:2398212818817494 32166165 10.1177/2398212818817494 PMC7058212

[CR13] Burnstock G, Kennedy C (1985) Is there a basis for distinguishing two types of P2-purinoceptor? Gen Pharmacol 16:433–440 2996968 10.1016/0306-3623(85)90001-1

[CR14] Burnstock G, Fredholm BB, Verkhratsky A (2011) Adenosine and ATP receptors in the brain. Curr Top Med Chem 11:973–1011 21401499 10.2174/156802611795347627

[CR15] Cao X, Li LP, Wang Q, Wu Q, Hu HH, Zhang M, Fang YY, Zhang J, Li SJ, Xiong WC, Yan HC, Gao YB, Liu JH, Li XW, Sun LR, Zeng YN, Zhu XH, Gao TM (2013) Astrocyte-derived ATP modulates depressive-like behaviors. Nat Med 19:773–777 23644515 10.1038/nm.3162

[CR16] Careaga M, Schwartzer J, Ashwood P (2015) Inflammatory profiles in the BTBR mouse: how relevant are they to autism spectrum disorders? Brain Behav Immun 43:11–16 24937468 10.1016/j.bbi.2014.06.006 PMC4776653

[CR17] Castora FJ (2019) Mitochondrial function and abnormalities implicated in the pathogenesis of ASD. Prog Neuropsychopharmacol Biol Psychiatry 92:83–108 30599156 10.1016/j.pnpbp.2018.12.015

[CR18] Cheng B, Zhu J, Yang T, Guo M, Lai X, Li Q, Chen J, Li T (2021) Vitamin A deficiency increases the risk of gastrointestinal comorbidity and exacerbates core symptoms in children with autism spectrum disorder. Pediatr Res 89:211–216 32225174 10.1038/s41390-020-0865-y

[CR19] Cheng B, Sun Q, Li X, Xiao M, Wei X, Wang S (2023) Vitamin A deficiency from maternal gestation may contribute to autistic-like behaviors and gastrointestinal dysfunction in rats through the disrupted purine and tryptophan metabolism. Behav Brain Res 452:114520 37268252 10.1016/j.bbr.2023.114520

[CR20] Chess S (1971) Autism in children with congenital rubella. J Autism Child Schizophr 1:33–47 5172438 10.1007/BF01537741

[CR21] Cohen MJ, Meador KJ, May R, Loblein H, Conrad T, Baker GA, Bromley RL, Clayton-Smith J, Kalayjian LA, Kanner A, Liporace JD, Pennell PB, Privitera M, Loring DW, Group NS (2019) Fetal antiepileptic drug exposure and learning and memory functioning at 6 years of age: the NEAD prospective observational study. Epilepsy Behav 92:154–164 30660966 10.1016/j.yebeh.2018.12.031

[CR22] El-Kordi A, Winkler D, Hammerschmidt K, Kastner A, Krueger D, Ronnenberg A, Ritter C, Jatho J, Radyushkin K, Bourgeron T, Fischer J, Brose N, Ehrenreich H (2013) Development of an autism severity score for mice using Nlgn4 null mutants as a construct-valid model of heritable monogenic autism. Behav Brain Res 251:41–49 23183221 10.1016/j.bbr.2012.11.016

[CR23] Ellul P, Maruani A, Peyre H, Vantalon V, Hoareau D, Tiercelin H, Rosenzwajg M, Klatzmann D, Delorme R (2023) Abnormal neutrophil-to-lymphocyte ratio in children with autism spectrum disorder and history of maternal immune activation. Sci Rep 13:22424 38104181 10.1038/s41598-023-49789-5 PMC10725503

[CR24] Ferrante A, Boussadia Z, Borreca A, Mallozzi C, Pedini G, Pacini L, Pezzola A, Armida M, Vincenzi F, Varani K, Bagni C, Popoli P, Martire A (2021) Adenosine A(2A) receptor inhibition reduces synaptic and cognitive hippocampal alterations in Fmr1 KO mice. Transl Psychiatry 11:112 33547274 10.1038/s41398-021-01238-5 PMC7864914

[CR25] Fredholm BB, Ap IJ, Jacobson KA, Klotz KN, Linden J (2001) International Union of Pharmacology. XXV. nomenclature and classification of adenosine receptors. Pharmacol Rev 53:527–552 11734617 PMC9389454

[CR26] Gilman SR, Iossifov I, Levy D, Ronemus M, Wigler M, Vitkup D (2011) Rare de novo variants associated with autism implicate a large functional network of genes involved in formation and function of synapses. Neuron 70:898–907 21658583 10.1016/j.neuron.2011.05.021 PMC3607702

[CR27] Guneykaya D, Ugursu B, Logiacco F, Popp O, Feiks MA, Meyer N, Wendt S, Semtner M, Cherif F, Gauthier C, Madore C, Yin Z, Cinar O, Arslan T, Gerevich Z, Mertins P, Butovsky O, Kettenmann H, Wolf SA (2023) Sex-specific microglia state in the Neuroligin-4 knock-out mouse model of autism spectrum disorder. Brain Behav Immun 111:61–75 37001827 10.1016/j.bbi.2023.03.023 PMC10330133

[CR28] Hirsch MM, Deckmann I, Santos-Terra J, Staevie GZ, Fontes-Dutra M, Carello-Collar G, Korbes-Rockenbach M, Brum Schwingel G, Bauer-Negrini G, Rabelo B, Goncalves MCB, Correa-Velloso J, Naaldijk Y, Castillo ARG, Schneider T, Bambini-Junior V, Ulrich H, Gottfried C (2020) Effects of single-dose antipurinergic therapy on behavioral and molecular alterations in the valproic acid-induced animal model of autism. Neuropharmacology 167:107930 31904357 10.1016/j.neuropharm.2019.107930

[CR29] Hooijmans CR, Rovers MM, de Vries RB, Leenaars M, Ritskes-Hoitinga M, Langendam MW (2014) SYRCLE’s risk of bias tool for animal studies. BMC Med Res Methodol 14:43 24667063 10.1186/1471-2288-14-43 PMC4230647

[CR30] Hornig M, Bresnahan MA, Che X, Schultz AF, Ukaigwe JE, Eddy ML, Hirtz D, Gunnes N, Lie KK, Magnus P, Mjaaland S, Reichborn-Kjennerud T, Schjolberg S, Oyen AS, Levin B, Susser ES, Stoltenberg C, Lipkin WI (2018) Prenatal fever and autism risk. Mol Psychiatry 23:759–766 28607458 10.1038/mp.2017.119 PMC5822459

[CR31] Horvath G, Otrokocsi L, Beko K, Baranyi M, Kittel A, Fritz-Ruenes PA, Sperlagh B (2019) P2X7 receptors drive poly(I:C) induced autism-like behavior in mice. J Neurosci 39:2542–2561 30683682 10.1523/JNEUROSCI.1895-18.2019 PMC6435822

[CR32] Jamain S, Quach H, Betancur C, Rastam M, Colineaux C, Gillberg IC, Soderstrom H, Giros B, Leboyer M, Gillberg C, Bourgeron T, Paris Autism Research International Sibpair S (2003) Mutations of the X-linked genes encoding neuroligins NLGN3 and NLGN4 are associated with autism. Nat Genet 34:27–29 12669065 10.1038/ng1136 PMC1925054

[CR33] Janesick A, Wu SC, Blumberg B (2015) Retinoic acid signaling and neuronal differentiation. Cell Mol Life Sci 72:1559–1576 25558812 10.1007/s00018-014-1815-9 PMC11113123

[CR34] Kaye AD, Allen KE, Smith IIIVS, Tong VT, Mire VE, Nguyen H, Lee Z, Kouri M, Baptiste CJ, Mosieri CN, Kaye AM, Varrassi G, Shekoohi S (2024) Emerging treatments and therapies for autism spectrum disorder: a narrative review. Cureus 16:e63671 39092332 10.7759/cureus.63671 PMC11293483

[CR35] Lewis MH, Rajpal H, Muehlmann AM (2019) Reduction of repetitive behavior by co-administration of adenosine receptor agonists in C58 mice. Pharmacol Biochem Behav 181:110–116 31054946 10.1016/j.pbb.2019.04.006 PMC6629027

[CR36] Li Z, Zhu YX, Gu LJ, Cheng Y (2021) Understanding autism spectrum disorders with animal models: applications, insights, and perspectives. Zool Res 42:800–824 34755500 10.24272/j.issn.2095-8137.2021.251 PMC8645879

[CR37] Lord C, Brugha TS, Charman T, Cusack J, Dumas G, Frazier T, Jones EJH, Jones RM, Pickles A, State MW, Taylor JL, Veenstra-VanderWeele J (2020) Autism spectrum disorder. Nat Rev Dis Primers 6:5 31949163 10.1038/s41572-019-0138-4 PMC8900942

[CR38] Meyza KZ, Blanchard DC (2017) The BTBR mouse model of idiopathic autism - current view on mechanisms. Neurosci Biobehav Rev 76:99–110 28167097 10.1016/j.neubiorev.2016.12.037 PMC5403558

[CR39] Meyza KZ, Defensor EB, Jensen AL, Corley MJ, Pearson BL, Pobbe RL, Bolivar VJ, Blanchard DC, Blanchard RJ (2013) The BTBR T+ tf/J mouse model for autism spectrum disorders-in search of biomarkers. Behav Brain Res 251:25–34 22958973 10.1016/j.bbr.2012.07.021 PMC3529977

[CR40] Napier M, Kumar A, Szulist N, Martin D, Scott AL (2024) P2X7 expression patterns in the developing Fmr1-knockout mouse hippocampus. Hippocampus 34:633–644 39269925 10.1002/hipo.23634

[CR41] Naviaux RK, Zolkipli Z, Wang L, Nakayama T, Naviaux JC, Le TP, Schuchbauer MA, Rogac M, Tang Q, Dugan LL, Powell SB (2013) Antipurinergic therapy corrects the autism-like features in the poly(IC) mouse model. PLoS ONE 8:e57380 23516405 10.1371/journal.pone.0057380 PMC3596371

[CR42] Naviaux JC, Schuchbauer MA, Li K, Wang L, Risbrough VB, Powell SB, Naviaux RK (2014) Reversal of autism-like behaviors and metabolism in adult mice with single-dose antipurinergic therapy. Transl Psychiatry 4:e400 24937094 10.1038/tp.2014.33 PMC4080315

[CR43] Ornoy A, Echefu B, Becker M (2023) Valproic acid in pregnancy revisited: neurobehavioral, biochemical and molecular changes affecting the embryo and fetus in humans and in animals: a narrative review. Int J Mol Sci. 10.3390/ijms25010390 38203562 10.3390/ijms25010390 PMC10779436

[CR44] Pugsley K, Scherer SW, Bellgrove MA, Hawi Z (2022) Environmental exposures associated with elevated risk for autism spectrum disorder may augment the burden of deleterious de novo mutations among probands. Mol Psychiatry 27:710–730 34002022 10.1038/s41380-021-01142-w PMC8960415

[CR45] Reynolds KE, Krasovska V, Scott AL (2021a) Converging purinergic and immune signaling pathways drive IL-6 secretion by Fragile X cortical astrocytes via STAT3. J Neuroimmunol 361:577745 34695768 10.1016/j.jneuroim.2021.577745

[CR46] Reynolds KE, Wong CR, Scott AL (2021b) Astrocyte-mediated purinergic signaling is upregulated in a mouse model of Fragile X syndrome. Glia 69:1816–1832 33754385 10.1002/glia.23997

[CR47] Reynolds KE, Huang E, Sabbineni M, Wiseman E, Murtaza N, Ahuja D, Napier M, Murphy KM, Singh KK, Scott AL (2024a) Purinergic signalling mediates aberrant excitability of developing neuronal circuits in the *Fmr1* knockout mouse model. Mol Neurobiol 61:9507–9528 38652351 10.1007/s12035-024-04181-w

[CR48] Reynolds KE, Napier M, Fei F, Green K, Scott AL (2024b) Dysregulated purinergic signalling in Fragile X syndrome cortical astrocytes. Neuromolecular Med 26:36 39254908 10.1007/s12017-024-08802-4

[CR49] Richter JD, Zhao X (2021) The molecular biology of FMRP: new insights into fragile X syndrome. Nat Rev Neurosci 22:209–222 33608673 10.1038/s41583-021-00432-0 PMC8094212

[CR50] Shaw KA, Williams S, Patrick ME, Valencia-Prado M, Durkin MS, Howerton EM, Ladd-Acosta CM, Pas ET, Bakian AV, Bartholomew P, Nieves-Munoz N, Sidwell K, Alford A, Bilder DA, DiRienzo M, Fitzgerald RT, Furnier SM, Hudson AE, Pokoski OM, Shea L, Tinker SC, Warren Z, Zahorodny W, Agosto-Rosa H, Anbar J, Chavez KY, Esler A, Forkner A, Grzybowski A, Agib AH, Hallas L, Lopez M, Magana S, Nguyen RHN, Parker J, Pierce K, Protho T, Torres H, Vanegas SB, Vehorn A, Zhang M, Andrews J, Greer F, Hall-Lande J, McArthur D, Mitamura M, Montes AJ, Pettygrove S, Shenouda J, Skowyra C, Washington A, Maenner MJ (2025) Prevalence and early identification of autism spectrum disorder among children aged 4 and 8 years - Autism and Developmental Disabilities Monitoring Network, 16 Sites, United States, 2022. MMWR Surveill Summ 74:1–22 40232988 10.15585/mmwr.ss7402a1 PMC12011386

[CR51] Suurvali J, Boudinot P, Kanellopoulos J, Ruutel Boudinot S (2017) P2X4: a fast and sensitive purinergic receptor. Biomed J 40:245–256 29179879 10.1016/j.bj.2017.06.010 PMC6138603

[CR52] Szabo D, Tod P, Goloncser F, Roman V, Lendvai B, Otrokocsi L, Sperlagh B (2022) Maternal P2X7 receptor inhibition prevents autism-like phenotype in male mouse offspring through the NLRP3-IL-1beta pathway. Brain Behav Immun 101:318–332 35065198 10.1016/j.bbi.2022.01.015

[CR53] Tessarollo L, Yanpallewar S (2022) TrkB Truncated isoform Receptors as transducers and determinants of BDNF functions 16. 10.3389/fnins.2022.847572 10.3389/fnins.2022.847572 PMC8934854 35321093

[CR54] Wang Q, Kong Y, Wu DY, Liu JH, Jie W, You QL, Huang L, Hu J, Chu HD, Gao F, Hu NY, Luo ZC, Li XW, Li SJ, Wu ZF, Li YL, Yang JM, Gao TM (2021) Impaired calcium signaling in astrocytes modulates autism spectrum disorder-like behaviors in mice. Nat Commun 12:3321 34059669 10.1038/s41467-021-23843-0 PMC8166865

[CR55] Whitehouse CM, Curry-Pochy LS, Shafer R, Rudy J, Lewis MH (2017) Reversal learning in C58 mice: modeling higher order repetitive behavior. Behav Brain Res 332:372–378 28624316 10.1016/j.bbr.2017.06.014 PMC5553316

[CR56] Wood AG, Nadebaum C, Anderson V, Reutens D, Barton S, O’Brien TJ, Vajda F (2015) Prospective assessment of autism traits in children exposed to antiepileptic drugs during pregnancy. Epilepsia 56:1047–1055 25963613 10.1111/epi.13007

[CR57] Wyatt LR, Godar SC, Khoja S, Jakowec MW, Alkana RL, Bortolato M, Davies DL (2013) Sociocommunicative and sensorimotor impairments in male P2X4-deficient mice. Neuropsychopharmacology 38:1993–2002 23604007 10.1038/npp.2013.98 PMC3746707

[CR58] Zhuang H, Liang Z, Ma G, Qureshi A, Ran X, Feng C, Liu X, Yan X, Shen L (2024) Autism spectrum disorder: pathogenesis, biomarker, and intervention therapy. MedComm 5:e497 38434761 10.1002/mco2.497 PMC10908366

[CR59] Zimmermann FF, Gaspary KV, Siebel AM, Leite CE, Kist LW, Bogo MR, Bonan CD (2017) Analysis of extracellular nucleotide metabolism in adult zebrafish after embryological exposure to valproic acid. Mol Neurobiol 54:3542–3553 27189619 10.1007/s12035-016-9917-z

[CR60] Zolkipli-Cunningham Z, Naviaux JC, Nakayama T, Hirsch CM, Monk JM, Li K, Wang L, Le TP, Meinardi S, Blake DR, Naviaux RK (2021) Metabolic and behavioral features of acute hyperpurinergia and the maternal immune activation mouse model of autism spectrum disorder. PLoS One 16:e0248771 10.1371/journal.pone.0248771 PMC7971557 33735311

